# Effects of unstable β-PheRS on food avoidance, growth, and development are suppressed by the appetite hormone CCHa2

**DOI:** 10.1080/19336934.2024.2308737

**Published:** 2024-02-19

**Authors:** Dominique Brunßen, Beat Suter

**Affiliations:** Institute of Cell Biology, University of Bern, Bern, Switzerland

**Keywords:** Drosophila, developmental delay, growth, roaming, satiety signaling

## Abstract

Amino acyl-tRNA synthetases perform diverse non-canonical functions aside from their essential role in charging tRNAs with their cognate amino acid. The phenylalanyl-tRNA synthetase (PheRS/FARS) is an α_2_β_2_ tetramer that is needed for charging the tRNA^Phe^ for its translation activity. Fragments of the α-subunit have been shown to display an additional, translation-independent, function that activates growth and proliferation and counteracts Notch signalling. Here we show in *Drosophila* that overexpressing the β-subunit in the context of the complete PheRS leads to larval roaming, food avoidance, slow growth, and a developmental delay that can last several days and even prevents pupation. These behavioural and developmental phenotypes are induced by PheRS expression in CCHa2^+^ and Pros^+^ cells. Simultaneous expression of β-PheRS, α-PheRS, and the appetite-inducing CCHa2 peptide rescued these phenotypes, linking this *β-PheRS* activity to the appetite-controlling pathway. The fragmentation dynamic of the excessive β-PheRS points to β-PheRS fragments as possible candidate inducers of these phenotypes. Because fragmentation of human FARS has also been observed in human cells and mutations in human *β-PheRS (FARSB)* can lead to problems in gaining weight, Drosophila *β-PheRS* can also serve as a model for the human phenotype and possibly also for obesity.

## Introduction

The cytoplasmic PheRS/FARS is a large and complex tRNA synthetase with a heterotetrameric structure consisting of two α and two β subunits. It charges the tRNA^Phe^ with its cognate amino acid phenylalanine (Phe). PheRS is conserved in all species throughout evolution [1]. The α-PheRS with the active site and the β-PheRS with the tRNA^Phe^ recognition site are only functional in aminoacylation in the complex [1]. In most cells, the two PheRS subunits stabilize each other [2]. Lower levels of one subunit result in a decrease in the other subunit [2,3] whereas increasing the expression of β-PheRS requires co-overexpression of α-PheRS [2,4].

Recently, Ho et al. [4] discovered an aminoacylation- and translation-independent function of *Drosophila* α-PheRS. Even a mutant α-PheRS with an inactivated active site for aminoacylation can accelerate growth and proliferation. Furthermore, an α-PheRS fragment that is present in some tissues counteracts Notch signalling in situations where this signal affects tissue homoeostasis by either promoting stem cell fate or differentiation [5].

Among the few reports describing possible non-canonical functions of vertebrate PheRS is a study performed in rats. *β-PheRS (FARSB)*, *isoleucyl-tRNA synthetase (IleRS)*, and *methionyl-tRNA synthetase (MetRS)* mRNA expression levels increase in spinal dorsal horn neurons upon peripheral nerve injury [6]. This suggested the possibility that FARSB, IARS, and MARS may act as neurotransmitters for transferring abnormal sensory signals after peripheral nerve damage [6].

The B5 domain of β-PheRS is not involved in aminoacylation but conserved through evolution, suggesting that it might provide a different, non-canonical, activity. Human B5 contains a ‘helix-turn-helix’ motive and two B5 domains hB5 and hB5×. With their particular distance from each other, these can bind DNA, forming a loop between them. The target DNA does not need to contain a specified motive but a specified length of 80 base pairs [7]. A related function as an mRNA binding protein was also suggested for PheRS after it was found in a screen as a possible mRNA binding protein [8].

The first human patients with mutations in FARSB were also described. Trans-heterozygous (also called compound heterozygous) mutations in FARSB were viable with severe health and growth problems [3,9,10]. The FARSB mutations caused growth restriction, brain calcification, and interstitial lung disease. The mutations in FARSB can also lead to lower protein levels of FARSB and its partner alpha subunit [3,9,10], but this did not seem to affect translation, suggesting that a non-canonical effect caused the clinical condition through an unknown mechanism [3].

In this study, we show that manipulating *Drosophila* β-PheRS levels and subunit expression can affect growth speed, the timing of pupation, and behavioural aspects, such as feeding and roaming. We also present evidence for the involvement of specific brain and/or intestinal cells in this process and we discuss the nature of the non-canonical activity β-PheRS.

## Results

### Two motives in the β-PheRS B5 domain are essential for viability

**Figure 1.**
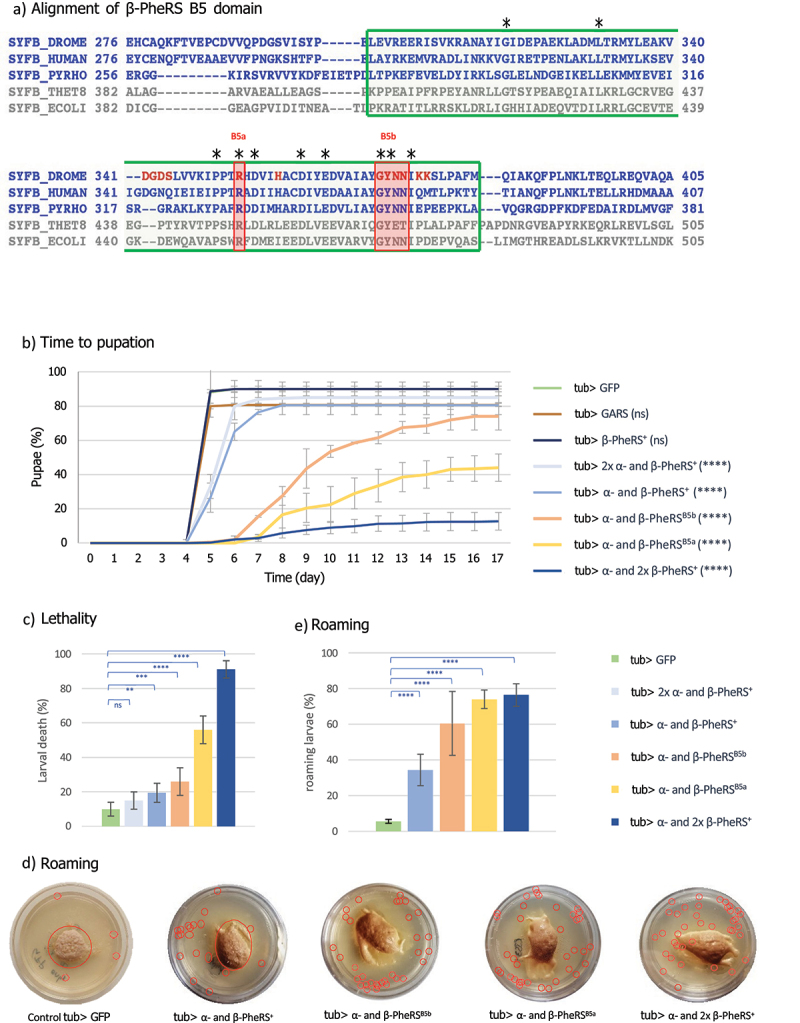
Structure and activity of the β-PheRS B5 domain. (a) Structure-based alignment of β-PheRS B5 domain and adjacent regions using the PROMALS3D method [11]. The sequences of prokaryotes and archaea/eukaryotes are shown in gray and blue, respectively. Amino acid residues labeled in red are predicted to bind DNA/RNA [7,13]. DROME, *Drosophila melanogaster*, human, *Homo sapiens*; PYRHO, *Pyrococcus horikoshii* ; THET8, *T. thermophilus*; ECOLI, *E. coli*. From [13]. Residues conserved over all species are marked with an asterisk. Marked with the green box is the B5 domain. Marked with the red box are the mutated sites B5a and B5b. The residue R^353^ and the motive GYNN^371–4^ were replaced by Alanines. (b) Time to pupation for the control, α-/β-PheRS^+^, α-/β-PheRS^B5a^, and α-/β-PheRS^B5b^ overexpressing larvae. One copy of the UAST-driven transgenes was used for each subunit unless indicated otherwise. 2× means two copies of the transgene were tested (homozygous transgene) (Mann-Whitney-U-Test). (c) Lethality caused by overexpression of α-/β-PheRS (Fisher’s exact test). (d) L3 larvae overexpressing GFP or different PheRS subunit combinations were tested for their effect on feeding, roaming, and survival on apple juice plates supplemented with yeast. The larvae are marked with red circles. (e) The percentage of roaming larvae was calculated (Fisher’s exact test). All experiments were performed in duplicates with 50–100 larvae. Graphs represent median ± SD. Indicated statistical tests were used to compare results to control. p-value not significant (ns) > 0.05, * ≤ 0.05, ** ≤ 0.01, *** ≤ 0.001, **** ≤ 0.0001.

While the functions of most PheRS domains are known, the biological function of the B5 domain of the β subunit (B5) is still unknown. B5 is not directly involved in aminoacylation but binds DNA and possibly also mRNA [7,8]. We mutated the codons for five conserved β-PheRS residues and motives because they might be important for DNA or RNA binding (Figure 1a, Table 1 [7,12,13]. Mutant *β-PheRS* transgenes under their native promoter were then tested in the *β-PheRS*^*null*^ background. Three of the five mutants fully rescued the mutant phenotype, indicating that they still provided sufficient activity to perform the essential functions of *β-PheRS*. These three mutants were not further analysed. The remaining two, *β-PheRS*^*B5a*^ and *β-PheRS*^*B5b*^ did not rescue, indicating that they were not functional (Table 1). This points to the R^353^ residue and the GYNN^371–4^ motive as essential for viability. Interestingly, these two sites are the most conserved ones (Figure 1a). Larvae containing either *β-PheRS*^*B5a*^ or *β-PheRS*^*B5b*^ in the *β-PheRS*^*null*^ background showed the same phenotype as the *β-PheRS*^*null*^ larvae. They hatched from the eggshell and appeared healthy. First instar larvae (L1 larvae) initially moved normally, but they did not grow and died during the first instar, a few hours after hatching. Survival to the larval stage is common for essential genes in *Drosophila*, where the maternal contribution of most gene products allows the development of embryos into larvae.

**Table 1.** Mutated residues & motives and the rescue ability of the mutated constructs.

Allele	Mutated Residues/Motives	Mutated to	Rescues *β-PheRS*^*null*^
B5a	R^353^	A	No
B5b	GYNN^371–4^	AAAA	No
B5c	DGDS^341–4^	AAAA	Yes
B5d	H^358^	A	Yes
B5e	KK^376–7^	AA	Yes

### Strong overexpression of α-/β-PheRS^+^, α-/β-PheRS^B5a^ or α-/β-PheRS^B5b^ leads to a developmental delay

An overexpression approach was used next. *α-PheRS, β-*PheRS^+^, *β-PheRS*^*B5a*^, and *β-PheRS*^*B5b*^ cDNAs were cloned behind the UAST promoter and overexpressed in the *α-/β-PheRS*^*+*^ background. Overexpressing β-PheRS^+^, β-PheRS^B5a^, and β-PheRS^B5b^ alone with the *actin-Gal4* or *tubulin-Gal4* (*tub-Gal4*) drivers did not lead to any phenotype. Similarly, no phenotype was seen when overexpressing them together with α-PheRS using the *actin-Gal4* driver. Interestingly, however, overexpression of the α-/β-PheRS^+^ subunits simultaneously with the strong *tub-Gal4* driver led to a slight developmental delay of 0–3 days. Surprisingly, this phenotype became much more pronounced when overexpressing the α- and the mutant β-PheRS^B5a^ or β-PheRS^B5b^ subunits simultaneously. In this case, a developmental delay of 2–12 days for β-PheRS^B5a^ and 0–11 days for β-PheRS^B5b^ was observed (Table 2).

**Table 2.** Time to pupation upon expression of α- and/or β-PheRS^X^ in the *α-/β-PheRS*^*+*^ background (in β-PheRS^X^, X stands for the wild-type (+) or the two mutant alleles *B5a* and *B5b*.).

Driver and Constructs UAST- β-PheRS^X^ Allele	*tub-Gal4*, *UAST-β-PheRS*^*X*^	*actin-Gal4*, *UAST-β-PheRS*^*X*^*and UAST-α-PheRS*	*tub-Gal4*, *UAST-β-PheRS*^*X*^*and* *UAST-α-PheRS*
β-PheRS^+^	normal	normal	Delay of 0–3 days
β-PheRS^B5a^	normal	normal	Delay of 2–12 days
β-PheRS^B5b^	normal	normal	Delay of 0–11 days

The time from the egg lay till the larvae started to pupate (time to pupation) was determined. It took the control larvae 5 days till half of the larvae had pupated. Upon overexpression of α-/β-PheRS^+^, α-/β-PheRS^B5b^, and α-/β-PheRS^B5a^, respectively, it took the larvae on average 6 days, 9 days, and 10 days, respectively (Figure 1b, Table 3).

**Table 3.** Median time till larvae pupated with tub-Gal4 overexpression and X-Gal80 inhibition.

Driver and inhibitor Constructs	*tub-Gal4*	*tub-Gal4* and *elav-Gal80*	*tub-Gal4* and *nSyb-Gal80*	*tub-Gal4* and *Su(H)GBE-Gal80*	*tub-Gal4* and *eye-Gal80*
GFP o/e	5	5	5	5	5
α- and β-PheRS^+^ o/e	6	5	5	5	6
α- and β-PheRS^B5b^ o/e	9	5	6	7	9
α- and β-PheRS^B5a^ o/e	10	5	6	7	9

Interestingly, overexpression of α-/β-PheRS^+^ with an additional *β-PheRS*^*+*^ construct (one α-construct and 2 *β-PheRS*^*+*^ constructs) induced 76% of the larvae to roam at day 4 and 91% of the collected L1 larvae died as larvae (Figure 1b, c). Roaming larvae wandered away from the food and many kept roaming and probably died due to starvation, but a fraction of them even crawled out of the dish and dried out (Figure 1d). Only 9% of the collected and counted L1 larvae reached the pupal stage within 5 to 12 days after egg-lay (Figure 1b). In contrast, overexpression of α-/β-PheRS^+^ with an additional *α-PheRS*^*+*^ construct induced a slightly milder developmental delay compared to overexpression of one copy each of α-/β-PheRS^+^ (Figure 1b). This indicates that β-PheRS and not α-PheRS is the main factor inducing the developmental delay.

Overexpressing α-/β-PheRS^B5a^ or α-/β-PheRS^B5b^ produced a stronger phenotype than the α-/β-PheRS^+^ overexpression but a less severe phenotype than the 1×α- and 2×β-PheRS^+^ overexpression. This might indicate that the B5 mutations make β-PheRS more active for this secondary activity and possibly less active for its canonical function.

### Overexpression of α-/β-PheRS induces roaming

*Drosophila* grows exclusively during the larval stages. During these 4 days, larvae grow ~ 200-fold from 0.01 mg to 2 mg through intense feeding during all stages [14]. Only once they have reached the optimal weight for pupation, do they stop feeding and wander away from the food. Larvae grown on apple juice plates supplemented with rich yeast paste in the centre stay mostly in the yeast [15]. Indeed, control larvae over-expressing GFP were mostly seen feeding on the yeast paste (Figure 1d) till the late L3 stage. In contrast, larvae overexpressing one copy each of α-/β-PheRS^+^, α-/β-PheRS^B5a^, or α-/β-PheRS^B5b^ tended to roam during all larval stages (Figure 1d). Whereas only 6% of the control larvae were roaming at day 4 (early L3 phase), larvae overexpressing α-/β-PheRS^+^, α-/β-PheRS^B5a^, and α-/β-PheRS^B5b^, respectively, were observed roaming in 34%, 60%, and 74% of the cases (Figure 1e).

### Overexpression of α-/β-PheRS decreases growth speed

**Figure 2.**
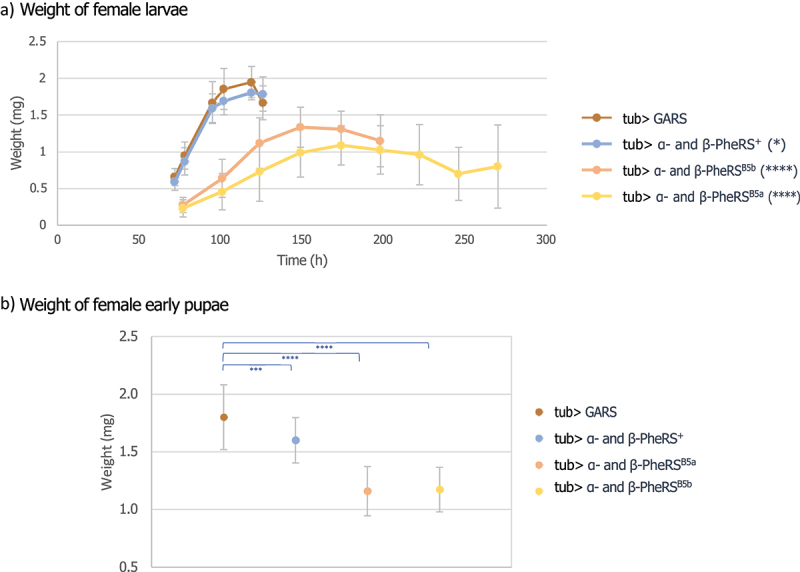
Weight of larvae and early pupae overexpressing PheRS subunits. (a) Weight development of control (tub> GARS) female larvae and female larvae overexpressing α-/β-PheRS^X^. Measurements were started on day 3 after egg-lay (Mann-Whitney-U-Test). (b) The pupal weight of control GARS overexpressing animals and α-/β-PheRS^X^ overexpressing animals was measured (one-way ANOVA). Graphs represent median ± SD, *n* = 50. Indicated statistical tests were used to compare results to control. p-value not significant (ns) > 0.05, * ≤ 0.05, **≤0.01, *** ≤ 0.001, ****≤ 0.0001.

Developmental delay can be caused by a failure to initiate pupation [16–19] or by reducing the growth rate [20]. To distinguish between the two possible mechanisms leading to a delay in pupation, the weight of individual larvae was measured upon α-/β-PheRS^+^, α-/β-PheRS^B5a^ or α-/β-PheRS^B5b^ (α-/β-PheRS^X^) overexpression. Even though we restricted the monitoring to the growth of female larvae, the time to pupation of the animals overexpressing α-/β-PheRS^B5a^ or α-/β-PheRS^B5b^ was very variable even between animals of the same genotype. Overexpression of glycyl-tRNA synthetase (GARS) was used as a control. Overexpression of α-/β-PheRS^+^ led to a small delay in growth and a difference in the max weight of 0.2 mg (7%) and the average pupal weight was 0.2 mg (11%) lighter (Figure 2), whereas overexpression of α-/β-PheRS^B5a^ or α-/β-PheRS^B5b^ led to a much more striking delay in growth. On average, these larvae reached their maximal weight 149 h and 174 h, respectively, after egg lay with a difference of 0.7 mg (32%) and 0.9 mg (44%), respectively, and 0.6 mg (35% and 36%) lighter pupae compared to the control (Figure 2). The growth is even more impaired in larvae overexpressing 1×α- and 2×β-PheRS^+^ (Fig. S1). Most of these larvae died as L3 larvae after they had stopped growing (Figure 1c). We conclude that the developmental delay is caused by reduced growth.

### Upon ubiquitous overexpression of PheRS, β-PheRS accumulates in IPCs

**Figure 3.**
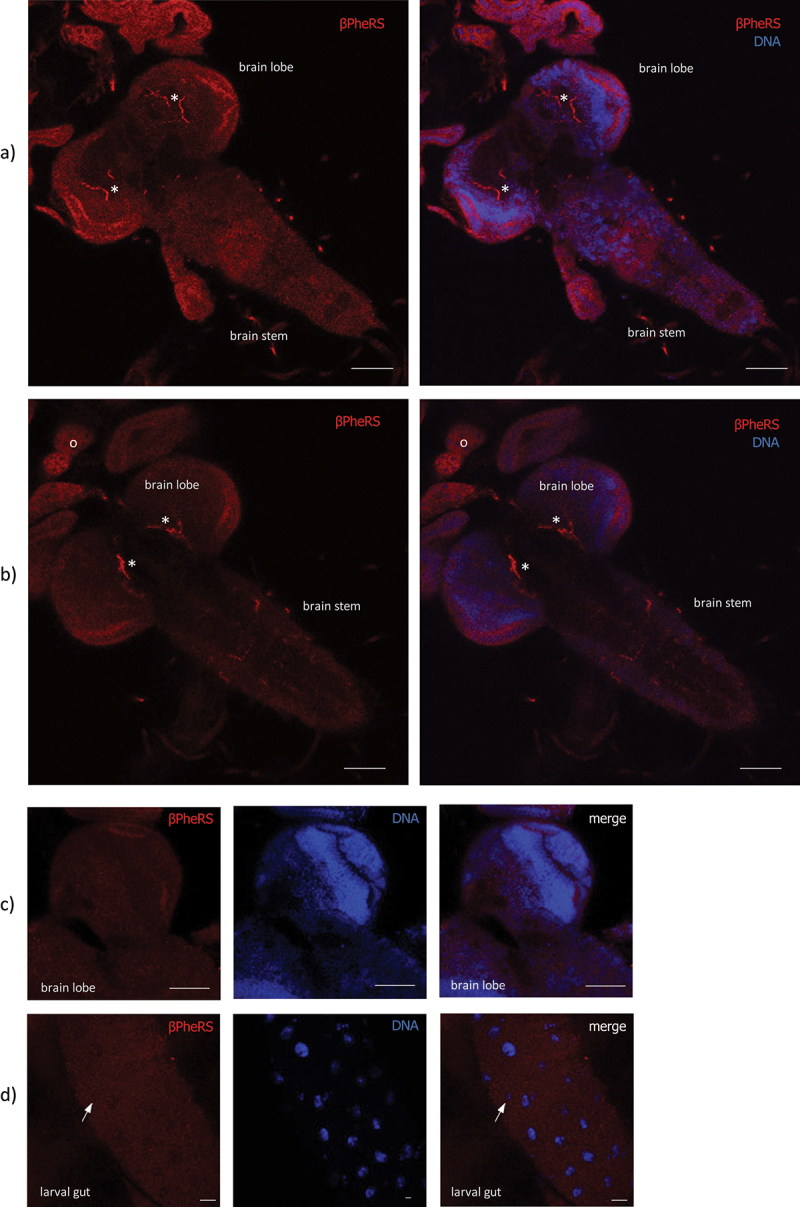
Accumulation pattern of β-PheRS upon GFP overexpression with *tub-Gal4* in larval tissue. (a, b) in the larval brain and the ring gland (o), β-PheRS does not accumulate above normal levels in specific cells. The extended thin fluorescent signals (*) stem from autofluorescence and are also seen in the control stainings without antibodies. Scale bar is 50 µm. (c) In the brain lobes, no accumulation of β-PheRS in specific neurons was observed. The scale bar is 50 µm. (d) In the gut, no accumulation is seen in the AMP clusters (arrow). The scale bar is 20 µm.

**Figure 4.**
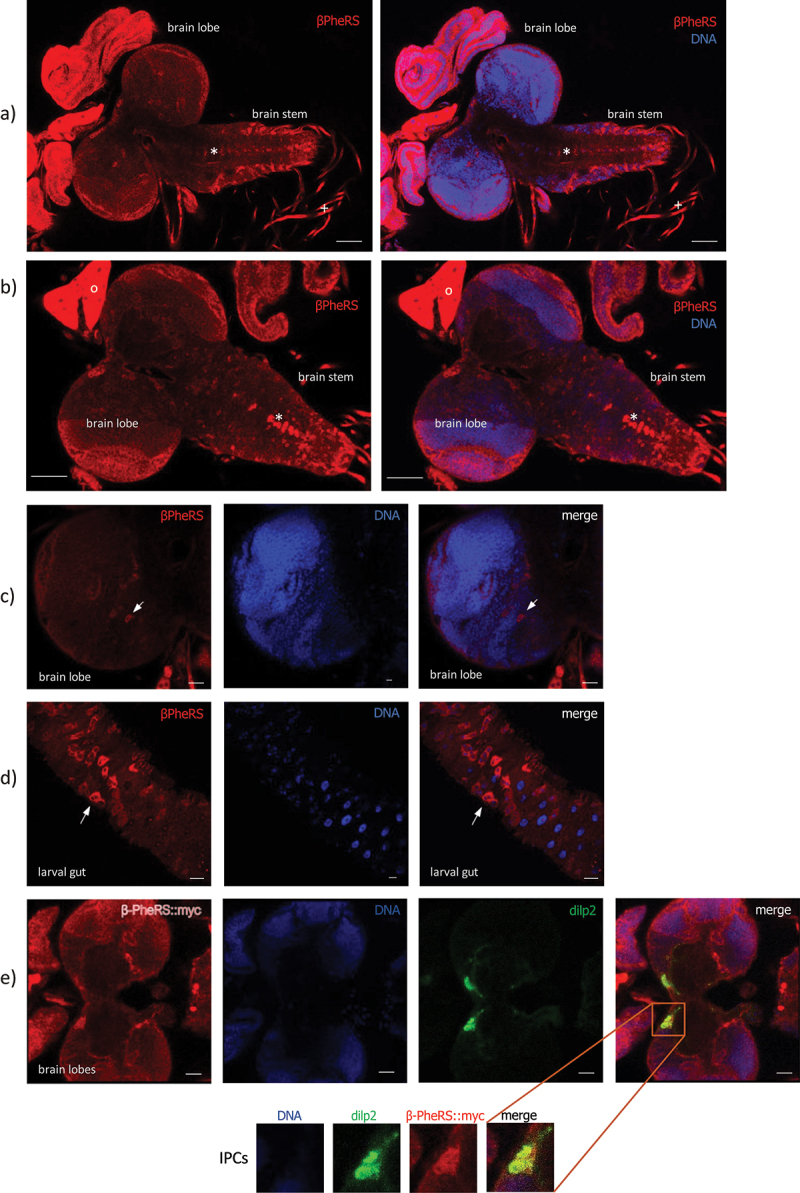
Accumulation pattern of β-PheRS upon α-/β-PheRS overexpression with *tub-Gal4* in larval tissues. In the larval brain, β-PheRS accumulates in the segmentally organized nerves (+ in a), in some not identified cells in the brain stem (* in a and b), and in the ring gland (o in b). The scale bar is 50 µm. (c) In the brain lobes, single neurons display elevated β-PheRS (arrow). The scale bar is 20 µm. (d) In the gut, high accumulation is seen in the AMP clusters (arrow). The scale bar is 20 µm. (e) In the brain lobes, IPCs display elevated β-PheRS. α-/β-PheRS:myc overexpressing brain lobes stained for Dilp2 and myc. Dilp2 marks the IPCs. The scale bar is 20 µm.

Control brains and guts overexpressing *tub-Gal4* driven GFP and stained for β-PheRS do not display uniform expression of β-PheRS (Figure 3). Upon overexpression of α-/β-PheRS with the ubiquitous driver *tub-Gal4*, the β-PheRS staining signal accumulated at higher levels only in a subset of tissues and cells while most of them showed the normal signal levels, pointing to an active and tight control mechanism that restricts cellular PheRS levels in most cells. Cells that do not implement the β-PheRS level control are the segmentally organized nerves (Figure 4a), the ring gland (Figure 4b), the brain lobes (Figure 4c), the AMP clusters in the larval gut (Figure 4d), and some cells in the brain stem (Figure 4a, b). Interestingly, a cluster of brain cells with higher staining levels co-stained with an anti-Dilp2 antibody, identifying these cells as Insulin-producing cells (IPCs) (Figure 4e). These cells might be candidates responsible for delaying larval growth and pupation.

### Tissue-specific inhibition of overexpression averts the developmental delay

**Figure 5.**
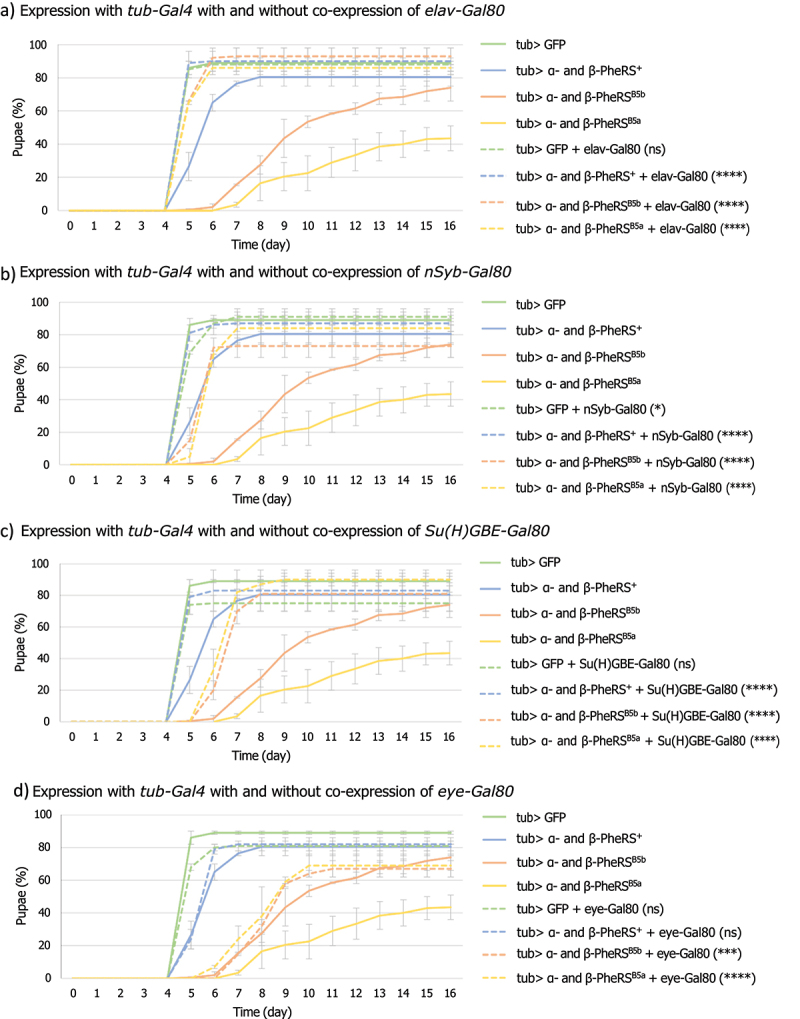
Overexpression of GFP or α-/β-PheRS^X^ with tubulin-Gal4 with or without the addition of a tissue-specific inhibitor of Gal4. (a) elav-Gal80, (b) nSyb-Gal80, (c) Su(H)GBE-Gal80, or (d) eye-Gal80 (Mann-Whitney-U-Test). All experiments were performed in duplicates with 50 larvae each. Graphs represent median ± SD. Mann-Whitney-U-Test was used to compare results to the control. p-value not significant (ns) > 0.05, * ≤ 0.05, ** ≤ 0.01, *** ≤ 0.001, **** ≤ 0.0001.

Different tissue-specific drivers were next used to try to narrow down candidate tissue(s) involved in the induction of the developmental delay by driving overexpression of α-/β-PheRS^X^. To facilitate a higher throughput testing, not the time to pupation but the time till the flies eclosed from the pupal case was determined in these experiments. With most drivers, the overexpression experiments did not lead to any developmental delay (Table 4 left column). We, therefore, needed to switch to inducing slow growth and developmental delay with the strong ubiquitous driver *tub-Gal4* and tested whether this effect can be repressed by tissue-specific repression through Gal80, the inhibitor of Gal4. In this way, α-/β-PheRS should not be overexpressed as much in the cells that express Gal80 and, if these cells are responsible for the slow growth during the larval stage, Gal80 expression in them would restore normal growth and development. Suppression of the delay by Gal80 should, therefore, identify the cells that express Gal80 as candidate cells and tissues where α-/β-PheRS overexpression causes the developmental delay. Two neuronal Gal80 drivers, the *elav-Gal80* and the *nSyb-Gal80* averted the developmental delay fully or to a high extent, with a remaining 1-day delay for the α-/β-PheRS^B5a^ or α-/β-PheRS^B5b^ overexpression (Figure 5a and b, Table 3). The *Su(H)GBE-Gal80* driver is among others reported to be expressed not only in the neuronal tissue but also in the PC cells in the larval gut [21]. This driver partially rescued the developmental delay down to 7 days instead of 9–10 days for α-/β-PheRS^B5a^ and α-/β-PheRS^B5b^ overexpression. Furthermore, it completely averted the developmental delay induced by α-/β-PheRS^+^ overexpression (Figure 5c and Table 3). In contrast, the control *eye-Gal80* driver did not avert the developmental delay caused by the overexpression of α-/β-PheRS^+^ and α-/β-PheRS^B5b^ but partially averted the developmental delay caused by the overexpression of the mutant α-/β-PheRS^B5a^ (Table 3). This partial effect was not further analysed in this study. The suppression of the developmental delay by neuronal and gut tissue-specific inhibition of overexpression (Table 3, Table S2) points to the potential roles of the brain and the gut in the induction of a developmental delay by α-/β-PheRS^X^.

**Table 4.** Cell- or Tissue-specific drivers tested for induction of a developmental delay.

Drivers used to overexpress α-/β-PheRS^X^			Drivers used to overexpress 1xα- and 2xβ-PheRS^X^		
Focus on Tissue/Cells (can additionally be expressed in other Tissue/Cells)	Gal4 driver	Delay	Focus on Tissue/Cells (can additionally be expressed in other Tissue/Cells)	Gal4 driver	Delay
Neuronal	*elav-Gal4*	no	Neuronal	*elav-Gal4*	no
Neuronal	*nSyb-Gal4*	no	Neuronal	*nSyb-Gal4*	yes
Specific neurons	*5OHO5-Gal4*	no	Specific neurons	*5OHO5-Gal4*	no
Optic lobe	*G124*^*c855a*^*-Gal4*	no	Optic lobe	*G124*^*c855a*^*-Gal4*	no
Eye	*ey-Gal4*	no	Eye	*longGMR-Gal4*	no
Fat body	*0.68Lsp2-Gal4*	no	Fat body	*0.68Lsp2-Gal4*	no
Neurons and Glia	*nrv2-Gal4*	no	Neuron and Glia	*nrv2-Gal4*	no
Motor neurons	*Hb9-Gal4*	no	Motor neuron	*Hb9-Gal4*	no
CCHa2^+^ cells	*CCHa2-Gal4*	no	CCHa2^+^ cells	*CCHa2-Gal4*	yes
Pros^+^ cells	*pros-Gal4*	no	Pros^+^ cells	*pros-Gal4*	yes
Wing	*wg-Gal4*	no			
Wing	*en-Gal4*	no			
Fat body	*ppl-Gal4*	no			
Fat body	*3.1LSP2-Gal4*	no			
Salivary gland	*fkh-Gal4*	no			
Ring gland	*phm-Gal4*	no			
Enterocytes	*NP1-Gal4*	no			
Stem cells	*delta-Gal4*	no			
AMP cells	*esg-Gal4*	no			
IPCs	*dilp2-Gal4*	no			
Ubiquitous	*actin-Gal4*	no			
Ubiquitous	*tub-Gal4*	yes			

### Tissue-specific expression of 1×α- and 2×β-PheRS^X^ identifies 3 cell types in which this causes growth delay

**Figure 6.**
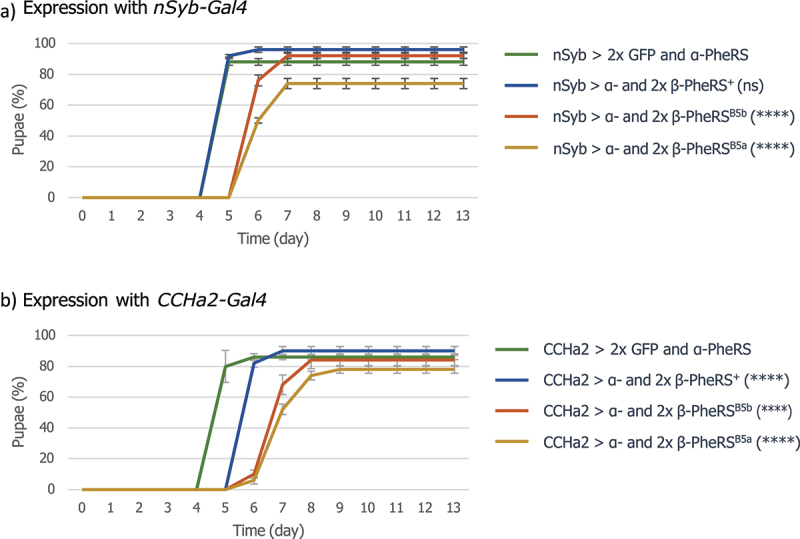
Effects of PheRS overexpression with *nSyb-* and *CCHa2-Gal4* drivers. Time to pupation when control GFP or 1×α- and 2×β-PheRS^X^ were overexpressed with the (a) *nSyb-Gal4* driver or (b) *CCHa2-Gal4* driver. All experiments were performed in triplicates with 50 larvae each. Graphs represent median ± SD. Mann-Whitney-U-Test was used to compare results to the control. p-value not significant (ns) > 0.05, * ≤ 0.05, ** ≤ 0.01, *** ≤ 0.001, **** ≤ 0.0001.

Adding to the α-/β-PheRS^+^ overexpression, a second copy of *UAST-β-PheRS*^*+*^ increased the severity of the phenotype drastically (Figure 1b-e). Several Gal4 drivers were used to test whether overexpression of one copy of *α-PheRS* together with two copies of *β-PheRS*^*X*^ delayed the eclosure of adult flies. Whereas many neuronal drivers did not lead to any developmental delay (Table 4 right column), the *nSyb-, CCHa2-*, and *pros-Gal4* drivers did. A time-to-pupation assay was subsequently also performed for these drivers. For the nSyb-Gal4 driver a developmental delay of 1 day was measured when this driver was used to overexpress 1×α- and 2×β-PheRS^B5a^ or 1×α- and 2×β-PheRS^B5b^ (Figure 6a). This positively identifies nSyb-Gal4^+^ cells as cells where overexpression of 1×α- and 2×β-PheRS^B5a^ or 1×α- and 2×β-PheRS^B5b^ elicits a developmental delay.

The *elav-Gal4* did not show a developmental delay while *nSyb-Gal4* induced one. The neuronal driver *nSyb-Gal4* is a stronger driver than *elav-Gal4* [22], but, additionally, the expression patterns of the two drivers differ, too (Figs. S2A and S2B). Furthermore, even though Gal4 drivers are often used as tissue-specific drivers, some of them express to some extent in other tissues as well. This was reported already by others [23,24] and observed by us. The neuronal *nSyb-Gal4* driver additionally showed expression in some enteroendocrine (EE) cells throughout the larval gut while *elav-Gal4* showed only expression in EE cells in a small unidentified part of the larval gut (Fig. S3A) and no expression in EE cells in the rest of the gut (Fig. S3B).

Overexpression of 1×α- and 2×β-PheRS^+^ with the *pros-Gal4* led to a developmental delay of 1 day and overexpression of 1×α- and 2×β-PheRS^B5a^ or 1×α- and 2×β-PheRS^B5b^ with the same driver to a delay of 4–7 days (Fig. S4A). This positively identifies the pros-Gal4^+^ cells as cells where overexpression of 1×α- and 2×β-PheRS^X^ elicits the developmental delay. Whether this is mediated by the Pros^+^ gut cells or Pros^+^ cells in another tissue cannot be deduced from these results. Even though the pros-Gal4 driver is one of the few drivers leading to a developmental delay when used to overexpress 1×α- and 2×β-PheRS^X^, it was less obvious how this would lead to mechanistic insights about the control of the developmental delay. We, therefore, focused our further analysis on a connection with CCHa2.

### Induction of the developmental delay in CCHa2^+^ cells

**Figure 7.**
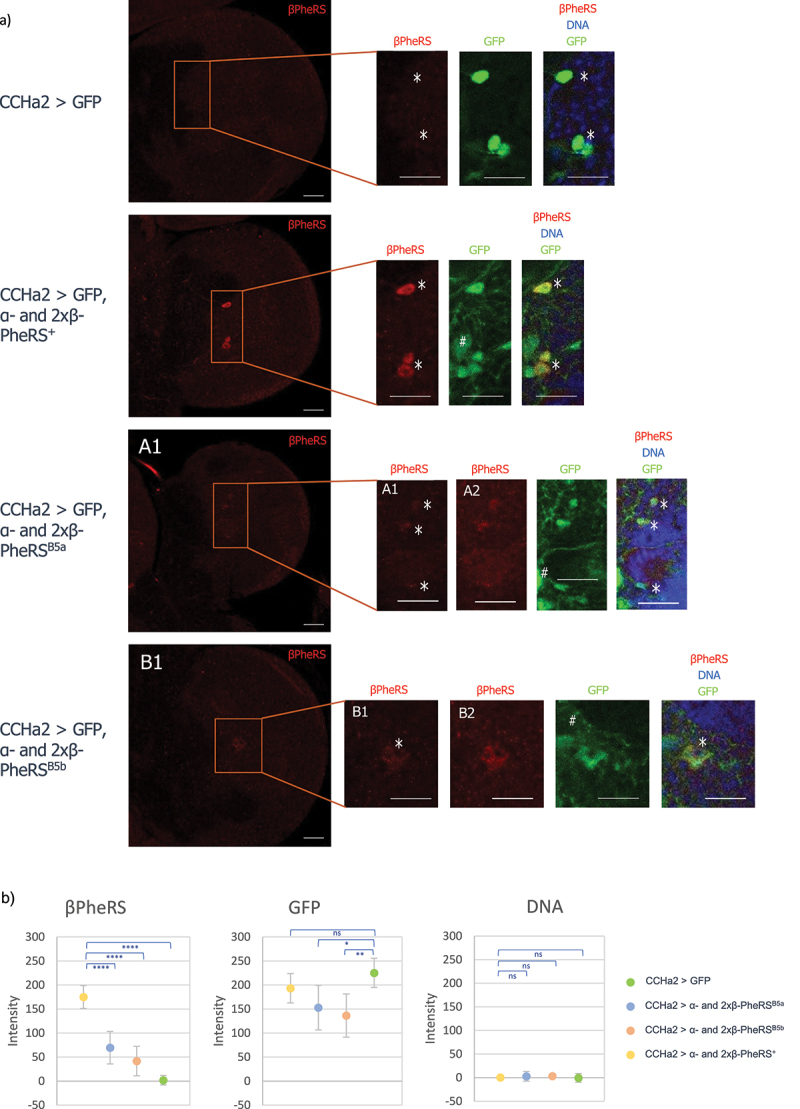
Effect of *CCHa2-Gal4* driven expression of GFP or 1×α- and 2×β-PheRS^X^. Larval brains were stained for β-PheRS and DNA (Hoechst). β-PheRS accumulates in some CCHa2>GFP^+^ cells (*). The background fluorescence in the green channel signal (#) stems from the landing platform used to insert the PheRS constructs. The scale bar is 25 µm. Pictures were taken with the same settings, except for A2 and B2, which are the same images as A1 and B1, respectively, taken with higher laser intensity. For the merged panels of A1 and B1, the red channel images taken with even higher laser power (A2 and B2 images) were used. Brain sizes differ slightly because of the variation in larval size due to their developmental delay. Note the reduced GFP signal in the α-/2×β-PheRS^X^ overexpressing micrographs compared to the UAS-GFP-only control. This seems to indicate that the *CCHa2* promoter becomes repressed upon α-/2×β-PheRS^X^ expression because the GFP signal serves as an activity reporter of the *CCHa2* promoter. The measurements in (b) show that the effect is reproducible. We note that the control with only UAS-GFP contains only UAS promoters driving GFP, the others have three additional UAS promoters to drive PheRS expression. On the other hand, the different PheRS over-expressing flies contain the same number of UAS constructs and can be compared directly. In these cases, the ones expressing the β-PheRS^B5a or B5b^ mutant protein show a stronger repressive effect on the *CCHa2* promoters driving the GFP expression (a, b), and they show the more severe developmental delay (Figure 6b). (b) The β-PheRS, GFP, and DNA signal intensity in the CCHa2^+^ neurons were measured compared to the surrounding background signal. The median ± SD for 10 neurons is shown. The wild-type β-PheRS accumulates stronger much than the B5 mutant protein and the GFP reporter is expressed at lower levels when the *β-PheRS*^*B5a*^ and *β-PheRS*^*B5b*^ mutants are expressed. Holm-Šidák’s multiple comparisons test was used to compare the results. p-value not significant (ns) > 0.05, * ≤ 0.05, ** ≤ 0.01, *** ≤ 0.001, **** ≤ 0.0001.

Neurosecretory cells and neuropeptides affect the growth rate and feeding behaviour in *Drosophila* [25,26], and activating or inhibiting expression of neuropeptides can change feeding behaviour as well as locomotion activity [27–32]. Driving α-/β-PheRS^X^ overexpression with the following drivers with demonstrated functions in satiety and starvation, *0098-Gal4*, *Dh44-Gal4*, *TH-Gal4*, *Hugin-Gal4*, *Dilp2-Gal4*, *NPF-Gal4*, *sNPF-Gal4*, *SIFa-Gal4*, *Taotie-Gal4*, and *AstA-Gal4*, did not lead to any delayed pupation. Furthermore, using an additional copy of *UAST-β-PheRS*^*x*^ with the *TH-Gal4* and *dilp2-Gal4* drivers did also not lead to delayed pupation. However, the *CCHa2-Gal4* driver led to a very weak delay in pupation when combined with α-/β-PheRS^X^ (one copy of β-PheRS^X^) and to a clear developmental delay when combined with 1×α- and 2×β-PheRS^X^ (Figure 6b). 1×α- and 2×β-PheRS^+^ overexpression led to a delay of 1 day in pupation onset while 1×α- and 2×β-PheRS^B5a^ or 1×α- and 2×β-PheRS^B5b^ overexpression led to 2 days delay (Table 5). Accumulation of β-PheRS in CCHa2^+^ cells was also observed under these conditions (Figure 7). This positively identifies *CCHa2-Gal4*^*+*^ cells as cells where overexpression of 1×α- and 2×β-PheRS^X^ elicits a developmental delay.

**Table 5.** Median time till larvae pupated when driven with nSyb- and CCHa2-Gal4.

	nSyb-Gal4	CCHa2-Gal4
GFP o/e	5	5
α- and 2xβ-PheRS^+^ o/e	5	6
α- and 2xβ-PheRS^B5a^ o/e	6	7
α- and 2xβ-PheRS^B5b^o/e	6	7

The *CCHa2-Gal4* driver is expressed in a subset of neurons in the brain (Figure 7, Fig. S2C), the gut (Fig. S3D), and the fat body (not shown [33]). The fat body-specific drivers *ppl-Gal4*, *0.68Lsp2-Gal4*, *3.1Lsp2-Gal4* did not lead to any developmental delay, and the tubulin-Gal4 driven delay was inhibited by elav-Gal80 expression (which should not affect the expression of Gal4 in the fat body). Therefore, we do not expect the fat body to be important for the developmental delay caused by overexpression of *CCHa2-Gal4*. The best candidates are therefore the CCHa2^+^ neurons and the CCHa2^+^ intestinal cells. We also note that overexpression of the β-PheRS^B5a/b^ mutant protein causes less accumulation of the β-PheRS protein signal in CCHa2^+^ cells than the wild-type overexpression. We will discuss this result and its implication in the next section and more extensively in the Discussion.

### Genetic interaction between PheRS and CCHa2

**Figure 8.**
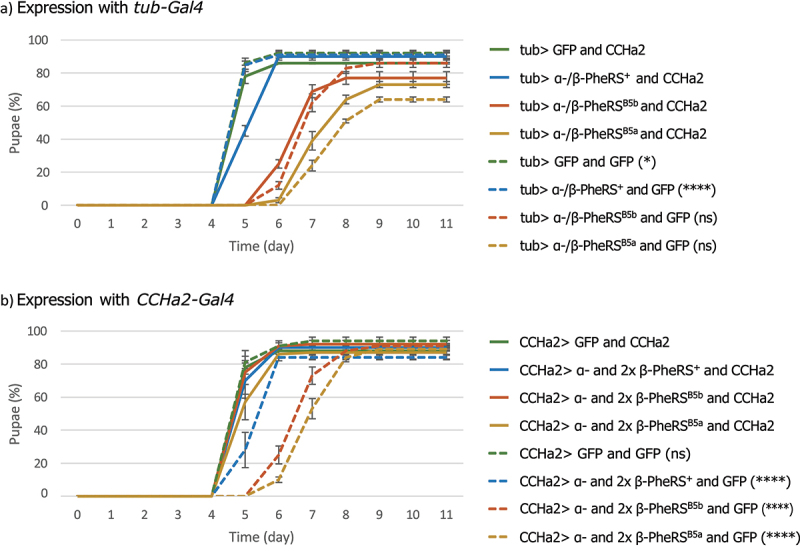
Rescuing the delay with CCHa2. (a) *tub-Gal4* driven overexpression of combinations of GFP, α-/β-PheRS^X^, and CCHa2. (b) *CCHa2-Gal4* driven overexpression of combinations of GFP, or 1×α- and 2×β-PheRS^X^, and CCHa2. All experiments were performed in triplicates with 50 larvae each. Graphs represent median ± SD. Mann-Whitney-U-Test was used to compare CCHa2 to GFP addition. p-value not significant (ns) > 0.05, * ≤ 0.05, ** ≤ 0.01, *** ≤ 0.001, **** ≤0.0001.

CCHa2 is an appetite-inducing peptide [28,34] and *CCHa2* mutants show reduced feeding activity and a delay in pupation of approximately 3 days [28]. Overexpression of α-/2×β-PheRS^X^ gives a similar phenotype as *CCHa2* mutants and overexpression α-/2×β-PheRS^X^ specifically in CCHa2^+^ cells (with the *CCHa2-Gal4* driver) led to a developmental delay. These closely related phenotypes led to the hypothesis that PheRS overexpression in CCHa2^+^ cells had some negative effect on *CCHa2* expression or activity and that the developmental delay of PheRS overexpression may be caused by this. The results seen in Figure 7 show that α-/2×β-PheRS^B5a/B5b^ overexpression has a repressive effect on the *CCHa2* promoter because the GFP reporter that is driven by the *CCHa2-Gal4* driver is weaker upon overexpression of α-/2×β-PheRS^B5a/B5b^ (Figure 7b). The further test whether PheRS overexpression represses the appetite by repressing *CCHa2* or competing with CCHa2 for a common downstream target, we additionally expressed the appetite inducing CCHa2 neuropeptide in larvae overexpressing also PheRS. In the first attempt, CCHa2 was co-overexpressed in larvae with α-/β-PheRS^X^ with the *tub-Gal4* driver (Figure 8a). In the control experiment, GFP was co-overexpressed with α-/β-PheRS^X^. This fairly ubiquitous overexpression of CCHa2 with α-/β-PheRS^+^ led to a slight developmental delay compared to GFP co-overexpression. This was unexpected and might also point to a negative effect of broad high-level overexpression of CCHa2 in tissues, where it is normally not expressed. Despite this possible negative effect of broad overexpression of CCHa2 with *tub-Gal4*, co-overexpression of CCHa2 with α-/β-PheRS^B5a^ or α-/β-PheRS^B5b^ showed a slight but not significant reduction of the developmental delay (Figure 8a).

To possibly reduce side effects from the broad over-expression of CCHa2 in all tissues, the *CCHa2-Gal4* driver was also used to overexpress 1×α- and 2×β-PheRS^X^ with the addition of a *UAST-CCHa2* construct or a *UAST-GFP* control construct. Co-expression of CCHa2 led to a full reduction in time to pupation compared to the control where GFP was co-overexpressed with 1×α- and 2×β-PheRS^X^ (Figure 8b). CCHa2 was fully able to avert the developmental delay when it was co-expressed. The presented results provide good evidence that the tissues and cell types that produce the developmental delay activity are the CCHa2+ cells in the nervous system and/or gut. The *CCHa2* rescue activity, on the other hand, might result from the higher expression of CCHa2 in any of the tissues where this driver is active, including the fat body.

Analogous experiments to test the effects of CCHa2 in Pros^+^ cells by expressing these constructs with the *pros-Gal4* driver did not show a reduction in time to pupation compared to the control where GFP was co-overexpressed (Fig. S4B).

### Protein levels upon overexpression of α-/β-PheRS^X^

Quantitative mass spectrometry analysis of L1 larvae overexpressing α-/β-PheRS^+^ with the tub-Gal4 driver in an *α-/β-PheRS*^*+*^ background showed an increase in α-PheRS and β-PheRS of 4.2× and 4.3×, respectively, with an α-PheRS/β-PheRS ratio of 1.02 (Table 6). In contrast, overexpression α-/β-PheRS^B5a^ or α-/β-PheRS^B5b^ in an *α-/β-PheRS*^*+*^ background led to an increase in β-PheRS of 2.0× and 2.1×, respectively, and an increase in α-PheRS levels of 2.6× . Therefore, overexpression of α-/β-PheRS^B5a^ or α-/β-PheRS^B5b^ led to a ratio of α-PheRS/β-PheRS^X^ of 1.3 and 1.2, respectively. These results show again that β-PheRS^B5a^ and β-PheRS^B5b^ are less stable, resulting in half the amount of stable protein upon overexpression compared to β-PheRS^+^. Due to the mutual stabilization of the subunits, we would expect an α-/β-PheRS^X^ ratio of 1. The increased ratio upon overexpression of α-/β-PheRS^B5a^ or α-/β-PheRS^B5b^ possibly results from similar synthesis levels as in α-/β-PheRS^+^, but higher turn-over of the less stable β-PheRS^B5a^ and β-PheRS^B5b^. This result confirms the observation that the CCHa2-driven overexpression leads to lower levels of mutant β-PheRS signal in the CCHa2+ neurons even though the overexpression of the mutant protein causes the stronger phenotypic effect. We will discuss the apparent correlation between the β-PheRS turnover, the levels of β-PheRS fragments, and the phenotypic effect in the next section.

**Table 6.** Protein levels in L1 larvae overexpressing α-/β-PheRS^X^ with the tub-Gal4 driver. (data are from table S4).

Gene	Protein	Protein level change (x) α/β-PheRS^+^ vs control	Protein level change (x) α/β-PheRS^B5a^ vs control	Protein level change (x) α/β-PheRS^B5b^ vs control
β-PheRS	Q9VCA5	4.2x	2.0x	2.1x
α-PheRS	Q9W3J5	4.3x	2.6x	2.6x
Ratio α-PheRS/β-PheRS		1.0	1.3	1.2

## Discussion

### The activity of β-PheRS and α-PheRS in delaying development

Mutating β-PheRS B5 domain residues and motives, respectively, R^353^ (B5a) and GYNN^371–4^ (B5b), respectively, led to lethality, indicating that they are essential or can at least not be replaced by alanine (Table 1). Because the expression levels of β-PheRS^B5b^ (and β-PheRS^B5a^, not shown) expressed under its endogenous promoter is much lower than the wild-type expression (Fig. S5) and the resulting phenotype indistinguishable from the *β-PheRS*^*null*^ mutant, the replacement of this residue or motive (see Figure 1a) by alanine causes the instability of β-PheRS. The other B5 domain mutations retained essential *β-PheRS* functions (Table 1), were viable, and their protein stable (Fig. S5 and not shown). The instability of the β-PheRS^B5a^ and β-PheRS^B5b^ mutant proteins was also evident upon their over-expression (Table 6; Table S4). In this case, the quantification was done by qMS.

To compensate for this instability, we attempted to overexpress *β-PheRS*^*X*^ and, because PheRS subunits are usually only stable if both subunits are overexpressed, we also overexpressed *β-PheRS*^*X*^ together with *α-PheRS*. This co-overexpression, but not the expression of either subunit alone, led to the roaming and developmental delay phenotype, suggesting that the formation of the α-*/*β-PheRS complex is a pre-requisite for the phenotype. Adding an additional copy of *UAS-α-PheRS to the UAS-α-PheRS/UAS-β-PheRS*, rescued the delay while adding a second *UAS-β-PheRS* turned out to extend the delay. This further suggests that the *β-PheRS* isoform produces the delay activity that induces roaming and food avoidance or prevents α-PheRS from stimulating feeding and growth.

In all cases tested, overexpression of α-/β-PheRS^B5a/B5b^ produced a stronger phenotype than α-/β-PheRS^+^ overexpression. This might indicate that the B5 mutations make β-PheRS more active for this secondary activity (and possibly less active for its canonical function). However, there is also evidence for alternative mechanisms. Overexpression of α-/β-PheRS causes only a mild hyperaccumulation in most cell types (Figure 4 [5, 13]; because most cells actively control the PheRS levels and cleave and degrade excessive PheRS. Because the B5 mutations cause additional instability of β-PheRS^B5a/B5b^ (Table 6, Table S4, Fig. S5, Fig. S6), the mutant β-PheRS^B5a/B5b^ seems to become fragmented even more. The cleavage of the two subunits is accompanied by the formation of stable proteolytic fragments [5] (Fig. S6). As detailed in the following paragraph, it is the more rapid or extensive fragmentation of β-PheRS^B5a/B5b^ that correlates with the severity of the developmental delay phenotype. This could indicate that a stable proteolytic fragment of β-PheRS might be the causative agent favouring roaming and developmental delay over feeding and growing. In this context, the wild-type R^353^ and GYNN^371–4^ sequences of β-PheRS are thus needed to stabilize β-PheRS and to reduce its fragmentation rate.

The mass spectrometry analysis of overexpressing α-/β-PheRS^+^ first instar larvae showed an approximate 2× higher accumulation of β-PheRS^+^ compared to β-PheRS^B5a/B5b^ despite the presence of the endogenous β-PheRS^+^ in the background (Table 6). Similarly, staining upon overexpression with the *CCHa2-Gal4* driver showed higher accumulation of β-PheRS^+^ than β-PheRS^B5a/B5b^ (Figure 7). Despite the lower levels, these α-/β-PheRS^B5a/B5b^ overexpressing larvae displayed a stronger delay phenotype, suggesting that the delay phenotype might be caused by a higher fragmentation because of their instability. There is precedent for aaRS fragments performing non-canonical activities [35–39]. Particularly relevant for this work is that the α-S fragment of the *Drosophila* α-PheRS subunit induces a proliferation phenotype and represses Notch activity [5].

### Non-cell-autonomous effect of α-/β-PheRS^X^ overexpression

dFOXO overexpression in whole flies suppresses growth and leads to roaming, and overexpression of dFOXO in wings or eyes reduces the size in the respective organ only, demonstrating that dFOXO acts in a cell-autonomous way [40]. This is not the case for α-/β-PheRS^X^ overexpression. Tissue-specific overexpression in the fat body, the eye- or wing disc did not decrease the size of the fat body, eye, or wing. There was also no change in the morphology of these organs apparent. Importantly, overexpression in all tissues simultaneously, combined with inhibition of expression in neuronal cells and the intestinal EE cells (*elav-Gal80*, *nSyb-Gal80*) averted most of the developmental delay. This points to the neuronal cells and the intestinal EE cells as possible sources of the non-cell-autonomous effect of overexpressed α-/β-PheRS^X^ in reducing growth and extending the larval L3 phase.

### Narrowing down the induction of the developmental delay to a few neuronal and/or gut cells

Overexpression of different combinations of α-/β-PheRS^X^ with *tub-Gal4*, *nSyb-Gal4*, *CCHa2-Gal4*, and *pros-Gal4* can lead to a developmental delay. Furthermore, co-expressing the inhibitor of Gal4, Gal80, as *elav-Gal80*, *nSyb-Gal80*, and *Su(H)-GBE-Gal80* reduced the developmental delay. If only one cell type induces the developmental delay, we expect that Gal4 and Gal80, respectively, are expressed in this cell type in all lines that show an effect. Should more than one cell type be able to induce the phenotype, the evaluation of the result becomes more complex. Starting with the first, simpler assumption, the expression patterns of these drivers led us to focus on the neuronal tissue and the gut (Table S2 and S3). *nSyb-Gal4* and *elav-Gal4* were used to express proteins neuronally and the *nSyb-Gal80* and *elav-Gal80* inhibitors were used to inhibit Gal4 drivers neuronally. Recent studies also described the activity of these drivers in the intestinal EE cells [23,24]. Chen et al. [23] discovered that *elav-Gal80* inhibits *AstA-Gal4* driven GFP expression in the CNS but also, and unexpectedly, in the EE cells. The same was true for the *nSyb-Gal80*. Chen et al. [23] described EE cell expression of *elav-Gal4* but no EE cell expression of the *nSyb-Gal4* line that they used. [23] described the expression pattern of two different *nSyb-Gal4* drivers and showed EE cell expression for one of the two *nSyb-Gal4* lines. We used the *nSyb-Gal4* line described in this paper as showing no EE cell expression but found that it also drove the expression of GFP in the EE cells (Fig. S3). From the intensity of the GFP expression signals, it appears likely that the *elav-Gal4* line expressed Gal4 too weakly to induce the developmental delay. The stronger *nSyb-Gal4* line induced a delay, albeit only a weak one. The *CCHa2-Gal4* and *pros-Gal4* lines reached a high enough Gal4 expression level in the important cells to induce the developmental delay. Both drivers show neuronal and gut expression. We have good evidence that the EE cells of the gut are at least not the only cause of the induction of a developmental delay. *Su(H)GBE-Gal80 is* expressed in some neuronal cells and the PC cells in the larval gut, but not in the intestinal EE cells of the larvae. Despite this, it was able to partially rescue the developmental delay. The fact that the rescue was only a partial one might point to some additive effects of the two tissues. Besides the unclear influence of the gut expression, we can conclude that all Gal4 lines that lead to a developmental delay and all Gal80 lines that rescue the developmental delay of *tub-Gal4* overexpression drive expression in the CNS. This strongly indicates an influence of the CNS, more specifically of the CCHa2^+^ and Pros^+^ cells of the CNS, in inducing a developmental delay when α-/β-PheRS^X^ are overexpressed.

Neurosecretory cells and neuropeptides are known to affect the growth rate, feeding behaviour, and locomotion activity in *Drosophila* [25–32]. IPCs are important regulatory cells for hunger and starvation response. Expression and release of DILP2, 3, and 5, as well as Drosulfakinin (DSK) in these specialized brain cells, regulate feeding and foraging behaviour [27]. DILP production in IPCs and their release from the cells are regulated by a variety of upstream factors such as neurosecretory cells and their respective neuropeptides. These neurosecretory cells and the release of their neuropeptides are in turn regulated by other factors sensing the nutritional state, food availability, and physiological state of the animal. Many factors involved in regulating hunger and satiety are known, but much remains unknown about the regulation of hunger, satiety, food-seeking, and food intake.

### Involvement of hunger and satiety signaling

IPCs and the ring gland are important tissues for the regulators of growth, maturation, and feeding [26,27]. This makes them candidates for providing a link to the α-/β-PheRS overexpression phenotypes of food avoidance, roaming, and developmental delay. Ubiquitous overexpression of α-/β-PheRS^X^ led to the accumulation of β-PheRS in the IPCs and the ring gland (Figure 4). We do not know whether this signal reflects higher levels of the tetrameric α-/β-PheRS or a stable β-PheRS fragment that is still recognized by the antibody. Testing for the effect of overexpressing α-/β-PheRS^X^ only in the IPCs with the *dilp2-Gal4* or in the ring gland with *phm-Gal4* did not lead to any developmental delay. Although not conclusive, this is again consistent with the notion that high levels of β-PheRS staining signals do not necessarily identify cells that produce the non-canonical β-PheRS activity. This observation is also consistent with the result that *CCHa2-Gal4* and *pros-Gal4* overexpressing larvae do not show enhanced accumulation of β-PheRS in IPCs even though they induce a developmental delay. The same is true for the ring gland accumulation of β-PheRS. Larvae overexpressing α-/β-PheRS^X^ with *tub-Gal4*, but having the expression blocked in the central nervous system by co-expression of *elav-Gal80*, still accumulate high β-PheRS levels in the ring glands (Fig. S7) and this does not lead to a developmental delay. β-PheRS accumulation in IPCs or the ring gland is, therefore, very unlikely to be the main cause of the developmental delay.

Overexpression of 1×α- and 2×β-PheRS^X^ with the *CCHa2-Gal4* driver leads to food avoidance and a developmental delay, indicating a possible involvement of hunger and satiety regulation in promoting the developmental delay. The CCHa2 neurons act upstream of the IPCs and are considered to link food availability to growth [27,33,41]. Overexpressing α-/β-PheRS in these neurons, therefore, appears to be the most likely mechanism for extending the larval phase. We would then expect that PheRS overexpression in CCHa2 neurons acts on their signalling to the IPCs in a manner that induces a food avoidance or a roaming phenotype. The additional expression of the appetite-inducing peptide CCHa2 in these 1×α- and 2×β-PheRS^X^ overexpressing larvae (with the CCHa2-Gal4 driver) averted the developmental delay. This is consistent with a possible appetite-reducing effect of α-/β-PheRS^X^ overexpression and a possible activity of α-/β-PheRS in modulating the CCHa2 signal. The reduced signal intensity of GFP upon α-/2×β-PheRS^B5a/B5b^ compared to α-/2×β-PheRS^+^ expression (Figure 7) points to a possible inhibitory effect of β-PheRS on the CCHa2 promoter. This possible mechanism needs to be verified and further investigated. While we cannot rule out an effect on translation, much would be difficult to explain by such an effect. For instance, we mapped the effect to the CCHa2^+^ cells in the brain or intestine, but not to the ones in the fat body which are thought to be the most important ones for CCHa2 signalling [33]. The Gal80 experiments showed no developmental delay when overexpressing α-/2×β-PheRS^X^ in almost all tissues except where elav-Gal80 inhibits it (Figure 5). This argues against a general interference with translation. The same is true for the result that α-/β-PheRS^+^ overexpression, which still provides translational activity, can delay growth and that the α-/2×β-PheRS^+^ has an even more severe effect on slowing down development. More consistent with the presented results is the hypothesis that an unidentified β-PheRS fragment is an important candidate activity (Fig. S6 and Fig. S8). Increasing production of β-PheRS fragments by different means (overexpression, destabilizing mutations) correlates with food avoidance and reduced growth. Because this phenotype is rescued by co-overexpression of CCHa2 in the same cells, it appears that high levels of one or more β-PheRS fragments might inhibit CCHa2 expression or its activity on a common downstream target. Preliminary evidence for the former has been presented (Figure 7). The significance of our findings may reach beyond *Drosophila* research. Fragmentation of PheRS (FARS) has also been observed in humans [42] and [43] and mutations in human *β-PheRS*, the *FARSB* gene, can lead to problems in gaining weight [3,9,10]. These mutations cause lower levels of FARSB to accumulate, too, indicating that the mutant FARSB is also destabilized. Because of the resemblance of the PheRS and FARS protein behaviour and growth problems of the destabilizing mutants in flies and man, the fly result presented here points to a potential mechanism for the human condition and to possible novel approaches to research ways to correct the balance between hunger and satiety signals by targeting *β-PheRS* in the context of obesity. Nevertheless, further studies on the mechanisms by which *β-PheRS* induces the behavioural effect (food avoidance) and the reduced weight gain (developmental delay) are still needed and the *Drosophila* research could again be useful for such studies.

## Materials and methods

### Material

**Key resource table**

**Table:** 

Reagent/Resource	Source	Identifier	Additional information
**Antibodies and dyes**			
Anti-β-PheRS	[2]	2B3	Immunostaining: 1:200 v/v
Anti-rabbit A647	Jackson ImmunoResearch Europe	111-605-144	1:400 v/v
Anti-mouse A647	Jackson ImmunoResearch Europe	115-606-146	1:400 v/v
Anti-rabbit A488	Life Technologies	A11008	1:400 v/v
Anti-dilp2	Gift from Hugo Stocker		1:400 v/v
Anti-myc	9E10	DSHB	Immunostaining: 1:4 v/v
Hoechst 33,342	Thermo Fisher Scientific	H3570

**Table:** 

Reagent/Resource	Source	Identifier	Additional information
**Fly stocks**			
Elav-Gal80	Gift from Alex Gould		Francis Crick Institute, United Kingdom
nSyb-Gal80	Gift from Hugo Stocker		ETH Zürich, Switzerland
Su(H)GBE-Gal80	Gift from I. Miguel-Aliaga, London, UK		[44]
Eye-Gal80	BDSC	#35822	
Tubulin (tub)-Gal4	BDSC	#5138	
actin-Gal4	BDSC	#4414	
Wingless (wg)-Gal4			
engrailed-Gal4	BDSC	#30564	
ey-Gal4 (longGMR-Gal4)	BDSC	#5535	
nSyb-Gal4	BDSC	#51635	
Nrv2-Gal4	BDSC	#6797	
G124^c855a^-Gal4	Gift from Boris Egger		University of Fribourg, Switzerland
pros-Gal4	BDSC	#80572	
Df7677	BDSC	#7677	
β-PheRS^a1103^	Walther 2010		
elav-Gal4	BDSC	#8760	
phm-Gal4	BDSC	#26159	
5OHO5-Gal4	Gift from Boris Egger		University of Fribourg, Switzerland
ppl-Gal4	[13]		
0.68Lsp2-Gal4	Gift from Raffael Koch		University of Geneva, Switzerland
3.1Lsp2-Gal4	Gift from Raffael Koch		University of Geneva, Switzerland
fkh-Gal4	BDSC	#78061	
NP1-Gal4	Gift from Hugo Stocker, ETH Zürich		ETH Zürich, Switzerland
delta-Gal4	BDSC	#45136	
esg-Gal4	Gift from Hugo Stocker		ETH Zürich, Switzerland
Hb9-Gal4	Gift from Soumya Banerjee		EPFL Lausanne, Switzerland
hts-Gal4	BDSC	#63463	
TH-Gal4 (ple-Gal4)	BDSC	#8848	
CCHa2-Gal4	BDSC	#84602	
AstA-Gal4	BDSC	#51979	
Taotie-Gal4 (Gr28b.b-Gal4)	BDSC	#57616	
0098-Gal4	BDSC	#77516	
SIFa-Gal4	BDSC	#84690	
Dilp2-Gal4	Gift from Hugo Stocker		ETH Zürich, Switzerland
Dh44-Gal4	BDSC	#84627	
NPF-Gal4	BDSC	#25682	
Hugin-Gal4	BDSC	#58769	
sNPF-Gal4	BDSC	#84706	
TM6B,Dfd-GMR-YFP, Sb, Tb	BDSC	#23232	
Sxl-Pe-eGFP	BDSC	#32565	
Yw; UAS-cyto-gars-myc/CyO	Gift from Albena Jordanova		VIB-U Antwerp Center for Molecular Neurology
Genomic β-PheRS^+^	[2]		
UAST-β-PheRS^+^	[2]		
UAST-α-PheRS	[2]		
UAST-CCHa2	Gift from Hiroko Sano		[33]
UAST-GFP	BDSC	#6658	
y, w, att2A[vas-phi]; attP-58A			[45]

**Table:** 

Reagent or Resource	Source	Identifier	Additional information
**Vectors**			
Genomic *β-PheRS*^*+*^ in *pw*^+^*SNattB*	[2]		Original vector from [46]
*β-PheRS*^*+*^ (cDNA) in *pUASTattB*	[2]		Original vector from [47]

**Table:** 

Reagent or Resource	Source	Identifier	Additional information
**Commercial assays and kits**			
Pierce® BCA Protein Assay kit	Thermo Scientific	23227

**Table:** 

Reagent or Resource	Source	Identifier	Additional information
**Software, algorithm**			
Leica Application Suite X (LAS X)	Leica		
GraphPad Prism	GraphPad		
Microsoft Excel	Microsoft		

**Buffers**

**Table:** 

**Fly food recipe** 20.4 l H_2_O 1.68 g Maize flour 720 g Yeast 1.8 g Syrup 192 g potassium sodium tartrate tetrahydrate 36 g Nipagin 120 ml propionic acid	**Apple juice plates** 1 l H_2_O tap 30 g Agar 350 ml Apple juice 35 g sugar 2 g nipagin	**10X PBS** 40 g NaCl, 1 g KCl, 7.2 g Na_2_HPO_4_, 1.2 g KH_2_PO_4_, pH adjusted to 7.4 filled up to 0.5 L
**PFA-fix** 1X PBSTT 4% Paraformaldehyde (w/v)	**PBST** 1X PBS 10X 0.2% Tween-20	**PBSTT** 1X PBST 0.1% TritionX-100
**Staining blocking solution** 1X PBSTT 5% non-fat dry milk (w/v)		

## Methods

### Fly keeping

Stocks in use were kept at 25°C in glass vials or plastic bottles with a day/night cycle (12 h/12 h). Larval experiments were performed at 25°C in a 24 h dark incubator. Stocks for long-term keeping were kept at 18°C in glass vials with a day/night (12 h/12 h) cycle on standard food.

### DNA constructs and generation of transgenic flies

The β-PheRS sequences stem from FlyBase. The genomic β-PheRS region cloned into the *pw*^*+*^*SNattB* transformation vector and the full-length cDNA cloned into the *pUAST-attB* transformation vector were obtained from [2]. The genomic and UAST constructs were mutated by site-directed mutagenesis, using the QuickChange® Site-Directed Mutagenesis Kit (Stratagene). The primers used for mutagenesis are listed in Table S1. All transgene constructs were verified by sequencing (Microsynth AG, Switzerland). Transgenic flies were generated using the *φ*C31-based integration system with the stock y, w, att2A[vas-phi]; attP-58A [45].

### Time to pupation assay and lethality

Egg lays were performed on day 0 between 9 am −1 pm on apple juice plates with yeast paste. 50 L1 larvae not displaying the Dfd-GMR-YFP signal from the balancer were collected on day 1 between 2–4 pm and placed onto new apple juice plates with yeast paste. The larvae were kept at 25°C in 24 h darkness. From day 4 on, every day at 5 pm the pupae were counted. The mean time to pupation was calculated with GraphPad Prism. The lethal larvae were determined as follows: collected larvae (50), minus the number of pupae, which were counted at the end of the pupation assay. All results represent biological duplicates or triplicates.

### Crosses for overexpression and its blocking in specific tissues

For all PheRS overexpression experiments, males containing the *tub-Gal4* driver and the *UAST-α-PheRS* construct (balanced over TM6B, *Dfd-GMR-YFP*, *Sb*, *Tb)* were crossed with the indicated UAST-β-PheRS^X^ stock and the selected offspring were used for the experiment. For the control experiments, males containing *tub-Gal4*/TM6B, *Dfd-GMR-YFP*, *Sb*, *Tb* were crossed with the UAST-control stocks indicated. For the Gal80 inhibition experiments, males containing the *tub-Gal4* and *UAST-α-PheRS* with the TM6B, *Dfd-GMR-YFP, Sb, Tb* balancer were crossed with females containing *UAST-βPheRS^X^* or *UAST-GFP* with the indicated tissue-specific-*Gal80* insert homozygous or over the TM6B, *Dfd-GMR-YFP*, *Sb*, *Tb* balancer. The offspring were then used as described in the ‘*Time to pupation assay and lethality’*. For all Gal4 experiments used for the pupation assays, males with a tested *Gal4* transgene either homozygous or over the balancer TM6B, *Dfd-GMR-YFP*, *Sb*, *Tb* were crossed to the females containing the indicated UAST-constructs. For all Gal4 experiments used for the time to adulthood experiments, the Gal4 males could be crossed to the UAST-containing females as they were obtained (with or without balancer) because the counterselection against the balancer was possible at the adult stage.

### Immunofluorescent staining and confocal microscopy

The tissue of interest was dissected in *PBS* (max 30 min) and fixed with *PFA-fix*. Wing discs and brains were fixed for 40 min and guts for 1 h. Fixed tissue was rinsed 3× and washed 3 × 10 min with *PBSTT* and blocked with *staining blocking solution* for 2 h at room temperature. The 1^st^ Ab was added to the *staining blocking solution* overnight at 4°C, rinsed 3×, and washed 3× for 20 min. Secondary antibodies were added in *staining blocking solution* for 4 hours at room temperature, rinsed 3×, and washed for 20 min. Hoechst 33,258 (5 µg/ml in PBST) was added for 20 min. The tissue was then washed again 2× for 20 min and mounted with Aqua/Poly Mount (Polysciences Inc., US). Image acquisition was performed on a Leica SP8 confocal microscope. The recipes of all solutions are noted in the *Key Resource Table*.

### Roaming assessment

Egg lays were performed on day 0 between 10 am −12 pm on apple juice plates with yeast paste. 50 L1 larvae not displaying the Dfd-GMR-YFP signal from the balancer were collected on day 1 between 2–4 pm and placed onto new apple juice plates with yeast paste. The larvae were kept at 25°C in 24 h darkness. On day 4 at 9 am, a picture was taken, and the roaming larvae were counted. All results represent biological duplicates.

### Larval weight development and pupal weight measurement

Egg lays were prepared on day 0 between 10 am −12 pm on apple juice plates with yeast paste. 4 hours later, the eggs with a Sxl-Pe-eGFP fluorescence signal were collected (female eggs were selected). 50 L1 larvae not displaying the Dfd-GMR-YFP signal from the balancer were collected on day 1 between 2–4 pm and put onto new apple juice plates with yeast paste. The larvae were kept at 25°C in 24 h darkness. From day 3 on, the larval weight was measured individually. The pupal weight was measured within the first 24 h after pupation. Statistical analysis was performed with GraphPad Prism.

### Measuring time to adulthood

Crosses were performed on standard food and the parental flies were transferred to new vials after two days. Two days later, they were removed from the second vials. Eclosed flies were sorted and counted every day for three days. If flies containing the Gal4 driver and flies containing the balancer eclosed from the first day on, the Gal4 driver was considered to not prolong the L3 phase.

### MS analysis

Egg laying was performed on apple juice plates without yeast for 2 hours. 26 hours later, 200 L1 larvae were collected without yeast contamination. The larvae were smashed in 100 µl urea buffer provided by the MS facility. Protein concentration was measured with the Pierce® BCA kit (Thermo Scientific) and the samples were analysed by the MS facility. Experiments were performed in biological triplicates.

Mass Spectrometry analysis by the MS facility: Smashed larvae were reduced, alkylated, and precipitated overnight at −20°C with five volumes of acetone. The pellet was re-suspended in 8 M urea/50 mM Tris/HCl pH 8.00 to a protein concentration of 1 mg/mL. Aliquots of 10 µg protein were double digested with LysC (Promega, ratio 1:100) for 2 hours at 37°C followed by Trypsin (Promega, ratio 1:100) at room temperature overnight. Digests were analysed in random order by loading 500ng onto a pre-column (C18 PepMap 100, 5 µm, 100A, 300 µm i.d. x 5 mm length) at a flow rate of 50 µL/min with solvent C (0.05% TFA in water/acetonitrile 98:2). After loading, peptides were eluted in back flush mode onto a home packed analytical Nano-column (Reprosil Pur C18-AQ, 1.9 µm, 120A, 0.075 mm i.d. x 300 mm length) using an acetonitrile gradient of 5% to 40% solvent B (0.1% Formic Acid in water/acetonitrile 4,9:95) in 180 min at a flow rate of 250nL/min. The column effluent was directly coupled to a Fusion LUMOS mass spectrometer (Thermo Fischer, Bremen; Germany) via a nano-spray ESI source. Data acquisition was made in data-dependent mode with precursor ion scans recorded in the orbitrap at a resolution of 120’000 (at m/z = 250) parallel to top speed fragment spectra of the most intense precursor ions in the linear trap for a cycle time of 3 seconds maximum. The HCD fragmentation type was applied for charge states 2 and 3, and ETD fragmentation for 4 to 9.

The mass spectrometry data was searched and quantified with MaxQuant [48] (version 1.5.4.1) using the *Drosophila melanogaster* uniprot (uniport) database [49] (release August 2017), to which common contaminants were added. The following parameters were used: digestion set to Trypsin/P, with a maximum of three missed cleavages; first search peptide tolerance set to 10 ppm, and MS/MS match tolerance to 0.4 Daltons. Carbamidomethylation on cysteine was given as a fixed modification; variable modifications were methionine oxidation, phenylalanylation, and protein N-terminal acetylation. Match between runs was not enabled. Protein intensities were reported as MaxQuant’s Label-Free Quantification (LFQ) values. Imputation and comparisons were performed for those protein groups for which there were at least 2 identifications in at least 1 group of replicates; re-normalization, filtering, and imputation were done with the DEP R package [50], using variance stabilization normalization [51] and ‘MinProb’ imputation method (draws from the 0.01th quantile). Differential expression tests were performed using empirical Bayes (moderated t-test) implemented in the R limma package [52]. The Benjamini and Hochberg [53] method was further applied to correct for multiple testing.

## Supplementary Material

Supplemental Material

## Data Availability

The authors confirm that the data supporting the findings of this study are available within the article and its supplementary materials.
