# Supplementary material for: Effects of unstable β-PheRS on food avoidance, growth, and development are suppressed by the appetite hormone CCHa2
Source: Fly (Austin). 2024 Feb 19;18(1):2308737. doi: 10.1080/19336934.2024.2308737 (PMC10880493; doi:10.1080/19336934.2024.2308737)
Supplement: Supplemental Material [file KFLY_A_2308737_SM4473.docx]

# Supplementary Tables and Figures

**Table S1: Primers used**

| Name | Sequence 5’ to 3’ |
| --- | --- |
| B5a-for | GTTAAGATCCCGCCCACAGCCCACGACGTCATCCACGCG |
| B5a-rev | CGCGTGGATGACGTCGTGGGCTGTGGGCGGGATCTTAAC |
| B5b-for | CGTGGCTATTGCCTACGCCGCCGCCGCCATTAAGAAGTCCCTGCC |
| B5b-rev | GGCAGGGACTTCTTAATGGCGGCGGCGGCGTAGGCAATAGCCACG |
| DGDS341A-for | TATCTGGAGGCCAAGGTCGCCGCCGCCGCCCTGGTGGTTAAGATC |
| DGDS341A-rev | GATCTTAACCACCAGGGCGGCGGCGGCGACCTTGGCCTCCAGATA |
| H358A-for | GCACGACGTCATCGCCGCGTGCGACATCTAC |
| H358A-rev | GTAGATGTCGCACGCGGCGATGACGTCGTGC |
| KK376A-fore | CTACGGCTACAACAACATTGCCGCCTCCCTGCCTGCATTCATGC |
| KK376A-rev | GCATGAATGCAGGCAGGGAGGCGGCAATGTTGTTGTAGCCGTAG |

**Table S2**: Gal80 inhibitor lines and their reported expression in larvae (Issigonis and Matunis 2010, Chen et al 2016, Weaver et al 2020). Red marked inhibitors suppressed the pupation delay if used to inhibit the effect of tubulin-Gal4 overexpression of α-/β-PheRSX. The listed Gal80 lines are expressed in the brain and the gut, but their cell type specific expression may differ.

|  | *tub-Gal4, elav- Gal80* | *tub-Gal4, nSyb- Gal80* | *tub-Gal4, Su(H)GBE-Gal80* | *tub-Gal4, eye-Gal80* |
| --- | --- | --- | --- | --- |
| Neurons/brain | ubiquitous | ubiquitous | some | eye-photoreceptor neurons |
| Gut | EE cells? | EE cells? | PCs | no |
| Fat body | no | no | no | no |
| Ring Gland | no | not assessed | no | no |

**Table S3:** Drivers and their expression patterns described in the literature (Sano et al 2015) and tested in this study with UAS-GFP expression. Green highlighted drivers led to a pupation delay if used to overexpress α-/β-PheRSX (tubulin-Gal4), 1xα- and 2xβ-PheRSX (nSyb-, CCHa2- or prospero-Gal4). Not highlighted elav-Gal4 did not induce a pupation delay. All drivers showed expression in the brain and the gut.

|  | *tub-Gal4* | *elav-Gal4* | *nSyb-Gal4* | *CCHa2-Gal4* | *pros-Gal4* |
| --- | --- | --- | --- | --- | --- |
| Neurons/brain | ubiquitous | ubiquitous | ubiquitous | some | some |
| Gut | ubiquitous | few EE cells | EE cells | EE cells | EE cells |
| Fat body | ubiquitous | no | no | Ubiquitous | no |
| Ring Gland | ubiquitous | no | partially | no | yes |

**Table S4:** qMS results for α-PheRS and β-PheRS

| Gene | Protein | LFQ_eBaye s log2-fold diff. α/β- PheRS vs control | LFQ_eBaye s adjpval α/β-PheRS vs control | LFQ_eBaye s log2-fold diff. B5a vs control | LFQ_eBaye s adjpval B5a vs control | LFQ_eBay es log2-fold diff. B5b vs control | LFQ_eBaye s adjpval B5b vs control |
| --- | --- | --- | --- | --- | --- | --- | --- |
| β-PheRS | Q9VCA5 | 2.081 | 2.6E-04 | 1.007 | 2.8E-03 | 1.084 | 3.0E-03 |
| α-PheRS | Q9W3J5 | 2.112 | 1.4E-04 | 1.349 | 1.3E-03 | 1.356 | 6.9E04 |
| Ratio α- PheRS/β- PheRS |  | 1.01 |  | 1.34 |  | 1.25 |  |

| Gene | Protein | LFQ_eBaye s log2-fold diff. B5a vs α/β-PheRS | LFQ_eBay es adjpval B5a vs α/β- PheRS | LFQ_eBaye slog2-fold diff. B5b vs α/β-PheRS | LFQ_eBay es adjpval B5b vs  α/β-PheRS |
| --- | --- | --- | --- | --- | --- |
| β-PheRS | Q9VCA5 | -1.074 | 3.0E-02 | -0.998 | 8.0E-02 |
| α-PheRS | Q9W3J5 | -0.763 | 6.9E-02 | -0.756 | 8.0E-02 |

Figure S1

2

1. 6

GFP


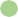

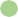

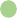

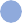

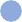

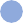

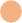

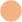

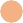

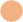

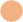

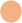

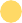

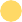

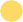

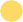

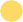

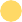

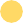

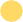

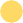

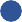

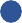

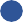

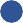

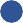

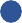

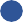

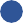

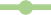

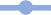

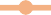

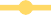

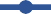


o/e: GFP

o/e: α- and β-PheRS+ (*)

o/e: α- and β-PheRSB5b (****)

o/e: α- and β-PheRSB5a (****) o/e: α- and 2x β-PheRS+ (****)

Weight (mg)

1. 2

PheRS (αβ) o/e

0. 8

PheRS (G371A) o/e

0. 4

PheRS (R353A) o/e

0

PheRS (αβ2) o/e

3 4 5 6 7 8 9 10 11

Time (day)

**Fig. S1: Weight development of mixed sex larvae.** Control larvae (o/e GFP) and larvae overexpressing α-/β-PheRSX. Measurements were started on day 3 after egg lay. Error bares show standard deviation. Graphs represent median ± SD, n = 20. Mann-Whitney-U-

Test was used to compare results to controls. p-value not significant (ns) > 0.05, * ≤ 0.05,

** ≤ 0.01, *** ≤ 0.001, **** ≤0.0001.

Figure S2

### A)


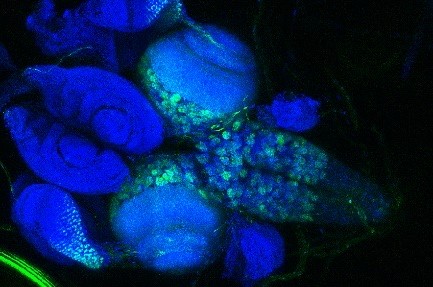


DNA


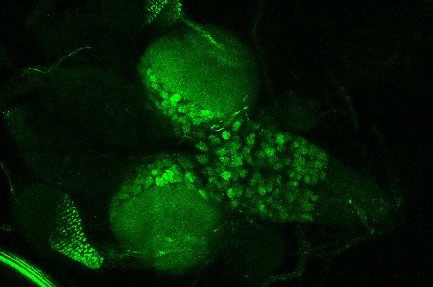


GFP

elav>GFP

### B)


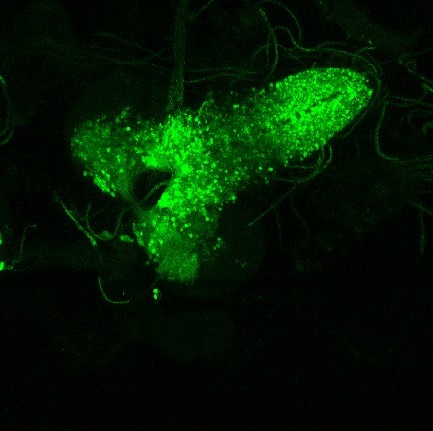


GFP


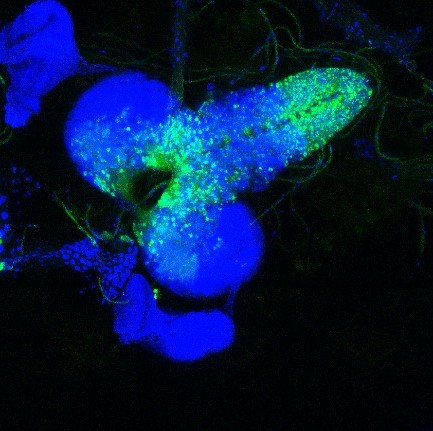


DNA

nSyb>GFP

C) CCHa2>GFP


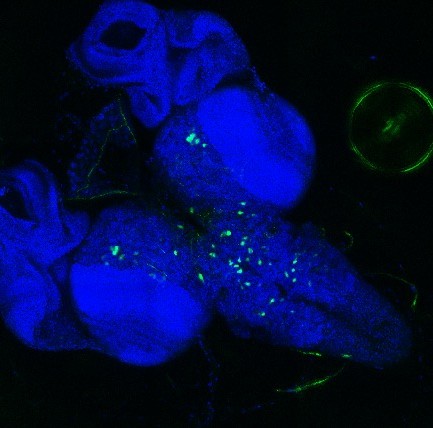


DNA


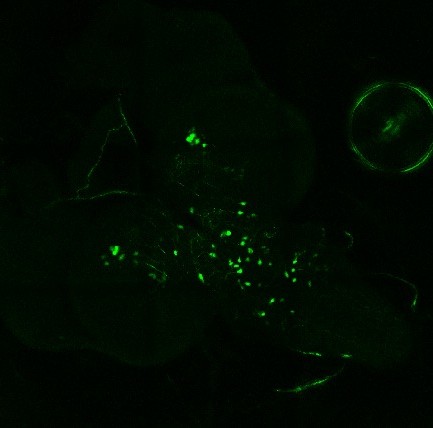


GFP

### D)


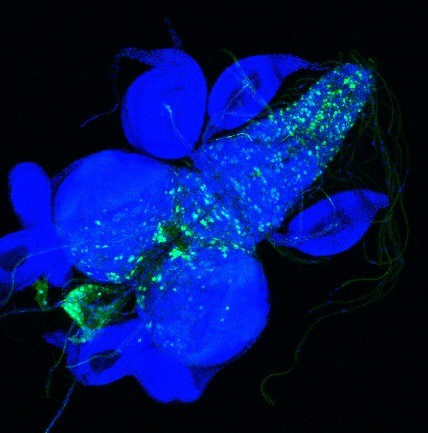


DNA


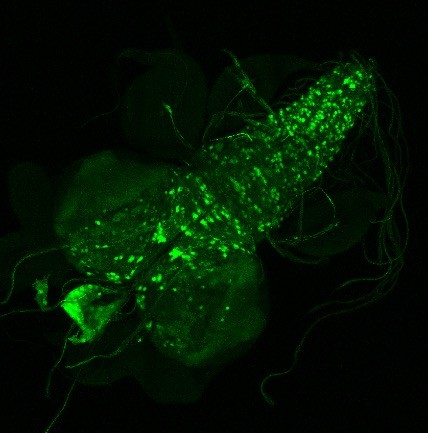


GFP

pros>GFP

**Fig. S2: Expression of Gal4 drivers in the larval brain revealed by UAS-GFP.** A) *elav-Gal4*,

B) *nSyb-Gal4*, C) *CCHa2-Gal4*, and D) *pros-Gal4*. Scale bar is 50 µm.

Figure S3

### A)


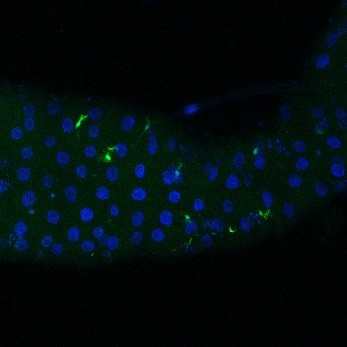


DNA


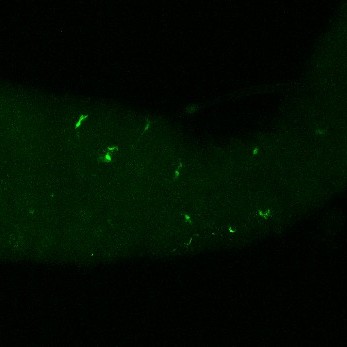


GFP

elav>GFP

### B)


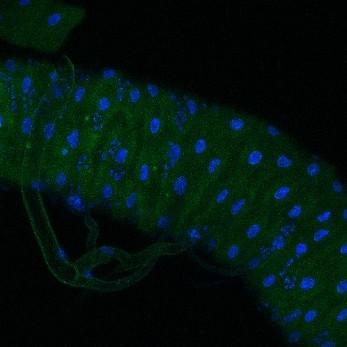


DNA


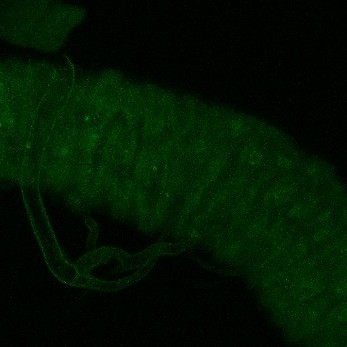


GFP

elav>GFP

- 1. ​


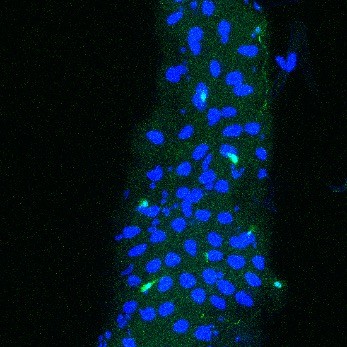


DNA


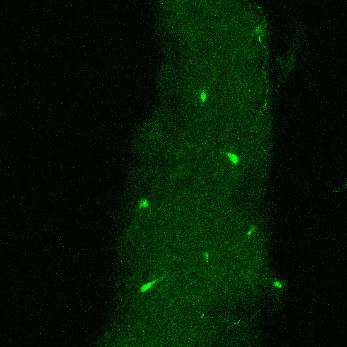


GFP

nSyb>GFP

- 1. CCHa2>GFP


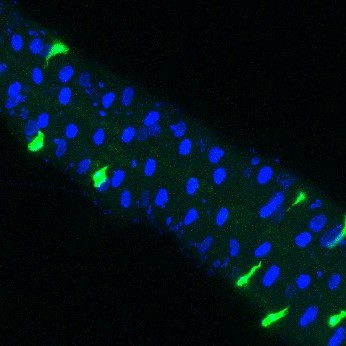


DNA


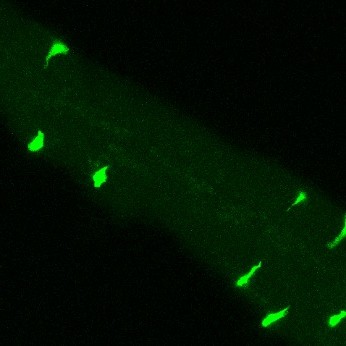


GFP

- 1. ​


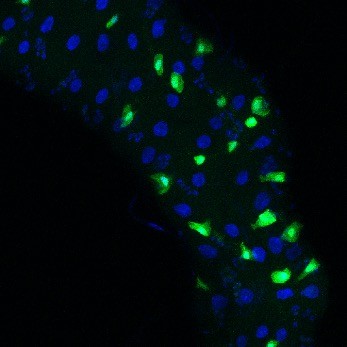


DNA


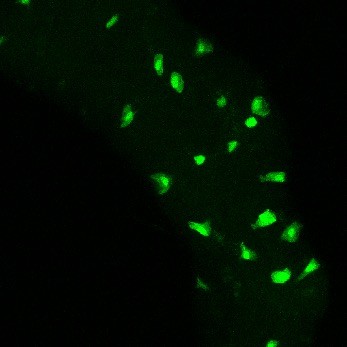


GFP

pros>GFP

**Fig. S3: Expression of Gal4 drivers in the larval gut revealed by UAS-GFP.** A-B) *elav- Gal4*, C) *nSyb-Gal4*, D) *CCHa2-Gal4* and E) *pros-Gal4*. Scale bar is 20 µm.

##### Figure S4

1. Expression of PheRS/GFP with *pros-Gal4*

100

GFP pros

pros > 2x GFP and α-PheRS

pros > α- and 2x β-PheRS+ (*****)

pros > α- and 2x β-PheRSB5b (****) pros > α- and 2x β-PheRSB5a (****)

80

βwt pros

Pupae (%)

60

G371A pros

40

R353A pros

20

0

0 1 2 3 4 5 6 7 8 9 10 11 12 13

Time (day)

1. Expression of PheRS/GFP and CCHa2 with *pros-Gal4*

100

GFP, CCHa2

pros> GFP and CCHa2

pros> α- and 2x β-PheRS+ and CCHa2 pros> α- and 2x β-PheRSB5b and CCHa2 pros> α- and 2x β-PheRSB5a and CCHa2 pros> GFP and GFP (ns)

pros> α- and 2x β-PheRS+ and GFP (*) pros> α- and 2x β-PheRSB5b and GFP (ns)

pros> α- and 2x β-PheRSB5a and GFP (ns)

80

CCHa2 beta wt

60

CCHa2 G371A

Pupae (%)

40

CCHa2 R353A

GFP, GFP

20

GFP, beta wt

0

GFP, G371A

0 1 2 3 4 5 6 7 8 9 10 11

Time (day)

GFP, R353A

**Fig. S4: Effects of overexpression of PheRS with pros-Gal4 and co-overexpression with CCHa2.** (A) Time to pupation when control GFP or 1xα- and 2xβ-PheRSX were overexpressed with the *pros-Gal4* driver. Overexpression of 1xα- and 2xβ-PheRS+ with the *pros-Gal4* led to a developmental delay of 1 day and overexpression of 1xα- and 2xβ-PheRSB5a or 1xα- and 2xβ- PheRSB5b with the same driver to a delay of 4-7 days. (B) *pros-Gal4* driven overexpression of GFP or α-/2xβ-PheRSX with GFP or CCHa2. The *pros-Gal4* driver slightly rescued the prolongation of the larval phase if CCHa2 was co-overexpressed with α-/2xβ-PheRS+, but it did not rescue significantly when CCHa2 was co-overexpressed with α-/2xβ-PheRSB5a or α-/2xβ-PheRSB5b. All experiments were performed in triplicates with 50 larvae each. Graphs represent median ± SD. Mann-Whitney-U- Test was used to compare results to control, respectively, CCHa2 to GFP addition. p-value not significant (ns) > 0.05, * ≤ 0.05, ** ≤ 0.01, *** ≤0.001, **** ≤0.0001.

##### Figure S5

*β*-*PheRS* genotype of the L1 larvae

null, *β-PheRS+*

con, β-PheRS+

null, *β-PheRSKK376-7A* con, *β-PheRSKK376-7A* null, *β-PheRSB5b*

con, *β-PheRSB5b*

null, *β-PheRSH358A*


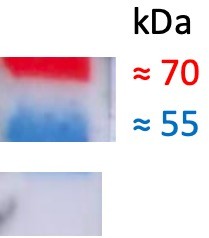


**Fig. S5: β-PheRS level in *β-PheRSnull* and rescue larvae.** The *β-PheRSnull* (null) compared to hemizygous *β-PheRS+* (con) is shown with or without the addition of a genomic β- PheRSX construct. The lower of the two bands is β-PheRS (marked with an arrow). The upper band is an unidentified cross reactivity that serves as a second loading control together with tS6K. *β-PheRSnull* larvae contain no visible β-PheRS anymore. The same is true for null larvae that also express the genomic construct *β-PheRSB5b*. Two other, viable point mutations in the B5 domain (*β-PheRSKK376-7A* and *β-PheRSH358A*; Fig. 1) still express the mutant protein. These mutants are viable. Reduced accumulation of the β-PheRSB5a and β- PheRSB5b mutant proteins was also observed by quantitative Mass Spectrometry of overexpressed PheRS (Table 6, Table S4) and with immunocytochemistry (Fig. 8).


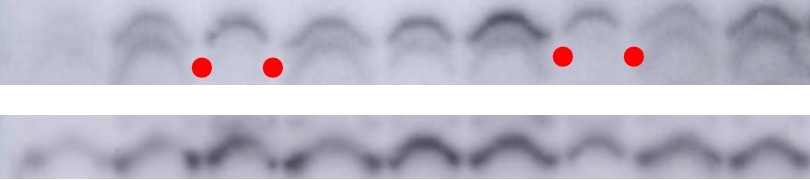


Antibody against

β-PheRS tS6K

null

con

Figure S6

*β*-*PheRS* genotype of the L1 larvae

tub > **1**xα-**2**xβ+-PheRS tub > **2**xα-**1**xβ+-PheRS tub > αβ+-PheRS

tub > GFP

####
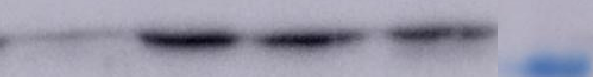
peIF2α


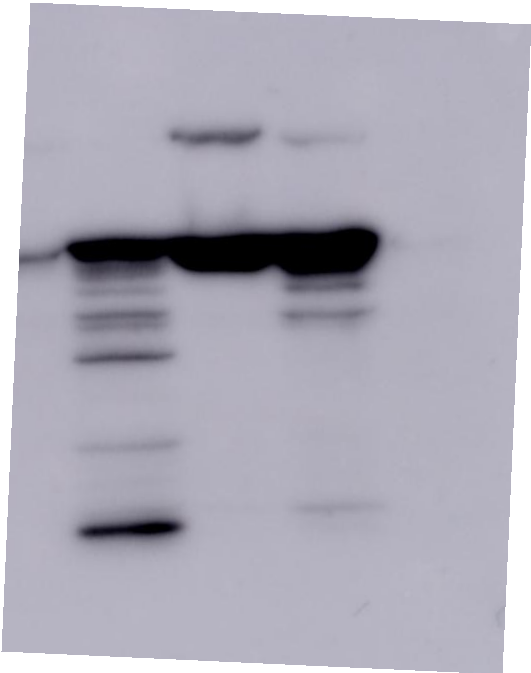

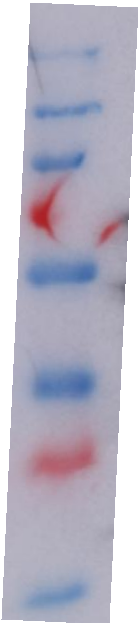

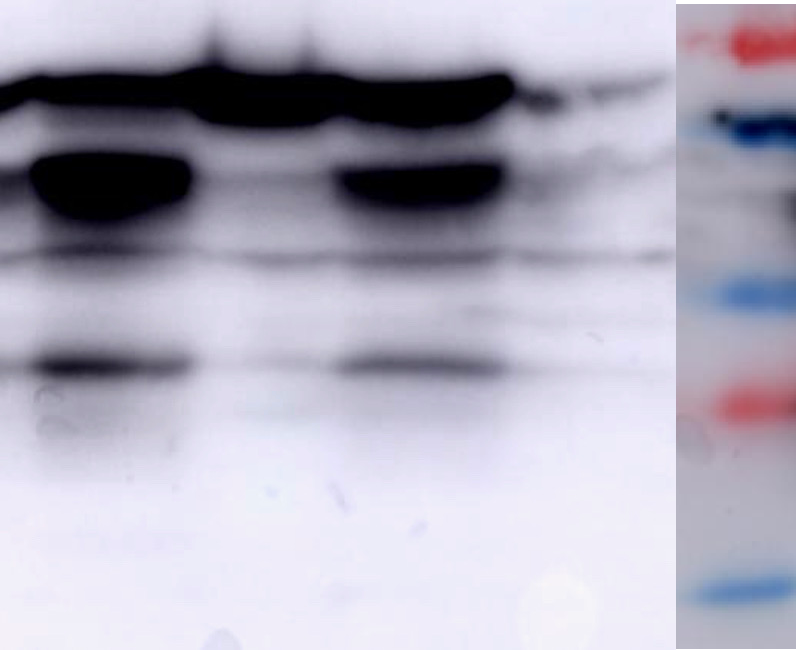


Antibody against

βPheRS

*

°

°

kDa 250

130

100

70

55

35

25

15

αPheRS

*

•

70

55

•

35

25

15

35

**Fig. S6: Western blot of 1xα, 2xβ-PheRS+; 2xα-, 1xβ-PheRS; 1xα-, 1xβ-PheRS, and GFP overexpressing larvae stained for β-PheRS and α-PheRS.** Marked (*) is the full-size protein. Marked with (°) are two β-PheRS fragments (20 kDa and 45 kDa) formed upon 1xα, 2xβ-PheRS+ overexpression, and marked with (•) are the two known α-PheRS fragments. The anti β-PheRS antibody was raised and purified against a peptide (C416-I429) from the β-PheRS B7 domain.

Figure S7

### A)


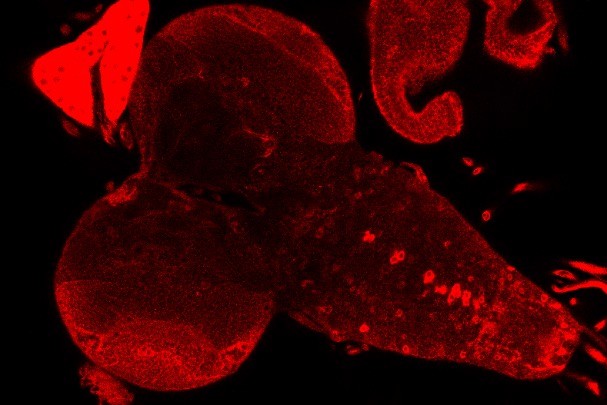


+

°

βPheRS

*

#


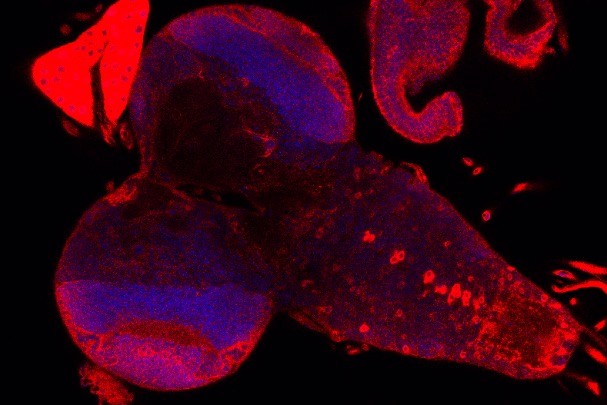


βPheRS

DNA

tub-Gal4 > α- and β- PheRS+

### B)


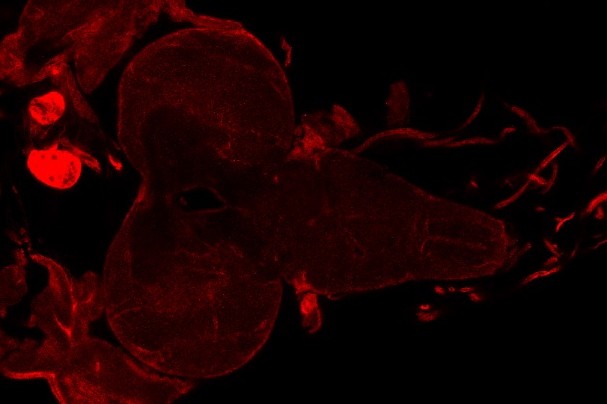


βPheRS

°

+

*

#


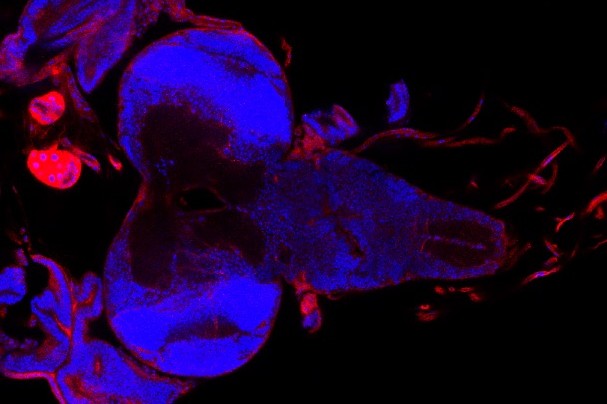


βPheRS DNA

tub-Gal4, elav-Gal80 > α- and β- PheRS+

1. ​


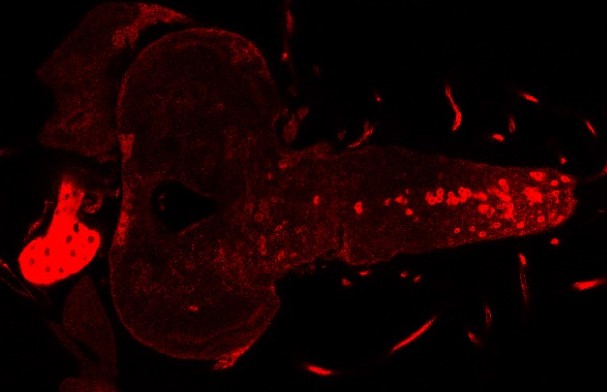


βPheRS

°

+

*

#


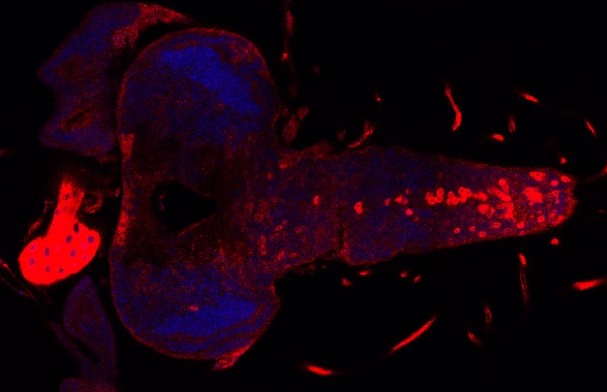


βPheRS DNA

tub-Gal4, Su(H)GBE-

Gal80 > α- and β- PheRS+

**Fig. S7: Change of β-PheRS levels (red) induced by overexpression with the *tub-Gal4* driver and partially inhibited by co-expression of Gal80 in different cells.** A) Accumulation pattern of β- PheRS upon overexpression of α-/β-PheRS+ with *tub-Gal4*. B)-C) Accumulation pattern of β-PheRS upon overexpression of α-/β-PheRS+ with *tub-Gal4* with co-expression of the Gal4 inhibitor Gal80 using B) *elav-Gal80* and C) *Su(H)GBE-Gal80*. Accumulation in the ring gland (+), in the IPCs (*), some other neurons (°), and in some cells in the brain stem (#) is seen with tubulin-Gal4 overexpression.

Upon inhibition with *elav-Gal80,* the brain did not show any increased accumulation of β-PheRS. Upon inhibition with *Su(H)GBE-Gal80*, the IPCs in the brain lobe and the cells in the brain stem still accumulated high levels of β-PheRS while the brain lobe (besides the IPCs) did not show an increased accumulation of β-PheRS. Scale bar is 50 µm.

Figure S8

Control larvae


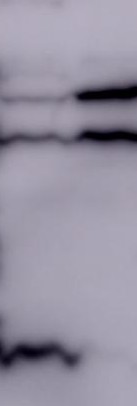


Antibody

against

β-PheRS

*

°

tub> α- and β- PheRS+ larvae

kDa 100

70

55

35

25

15

100

70

55


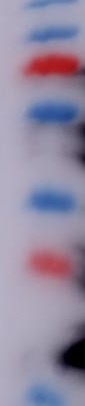


##
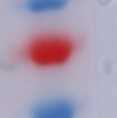

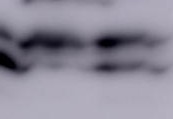
hts

kDa 70

rich food standard food

Feeding conditions

55


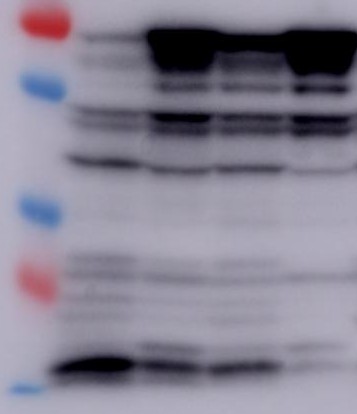


Antibody

against

* β-PheRS

°

°

rich food Standard food refed

starved

Feeding conditions

35

25

15


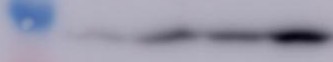
35

## Gapdh

**Fig. S8: Western blot of food dependent changes of β-PheRS.** Control and α-/βPheRS+ overexpressing larvae were raised on rich food (yeast) or standard food. Larvae raised on rich food showed an accumulation of an approximately 20 kDa β-PheRS fragment and a decrease of full size β-PheRS compared to larvae raised on standard food. In α-/β-PheRS+ overexpressing larvae, starvation reduced this 20 kDa fragment to a lower level than in larvae raised on standard food (or rich food). In α-/β-

PheRS+ overexpressing larvae, a second fragment of approximately 45 kDa showed a similar abundance pattern in terms of food dependence and starvation as the 20 kDa fragment. The anti- β-PheRS antibody was raised and purified against a peptide (C416-I429) from the β-PheRS B7 domain. Accordingly, this staining only reveals fragments containing this B7 peptide. Other β-PheRS fragments are not visualized but may be responsible for the growth reduction.
